# Sharing administrative health data with private industry: A report on two citizens' juries

**DOI:** 10.1111/hex.13268

**Published:** 2021-05-28

**Authors:** Jackie Street, Belinda Fabrianesi, Carolyn Adams, Felicity Flack, Merran Smith, Stacy M. Carter, Sean Lybrand, Anthony Brown, Serena Joyner, Judy Mullan, Luise Lago, Lucy Carolan, Katie Irvine, Coralie Wales, Annette J. Braunack‐Mayer

**Affiliations:** ^1^ Australian Centre for Health Engagement, Evidence and Values (ACHEEV), School of Health and Society University of Wollongong Wollongong NSW Australia; ^2^ Macquarie Law School Macquarie University Sydney NSW Australia; ^3^ Population Health Research Network University of Western Australia Perth WA Australia; ^4^ Amgen Australia Pty Ltd. North Ryde NSW Australia; ^5^ Health Consumers NSW Sydney NSW Australia; ^6^ Centre for Health Research Illawarra Shoalhaven Population University of Wollongong Wollongong NSW Australia; ^7^ The Centre for Health Record Linkage North Sydney NSW Australia; ^8^ Western Sydney Local Health District North Parramatta NSW Australia

**Keywords:** big data, community participation, consent, deliberative methods, health data, private sector

## Abstract

**Background:**

There is good evidence of both community support for sharing public sector administrative health data in the public interest and concern about data security, misuse and loss of control over health information, particularly if private sector organizations are the data recipients. To date, there is little research describing the perspectives of informed community members on private sector use of public health data and, particularly, on the conditions under which that use might be justified.

**Methods:**

Two citizens' juries were held in February 2020 in two locations close to Sydney, Australia. Jurors considered the charge: ‘Under what circumstances is it permissible for governments to share health data with private industry for research and development?’

**Results:**

All jurors, bar one, in principle supported sharing government administrative health data with private industry for research and development. The support was conditional and the juries' recommendations specifying these conditions related closely to the concerns they identified in deliberation.

**Conclusion:**

The outcomes of the deliberative processes suggest that informed Australian citizens are willing to accept sharing their administrative health data, including with private industry, providing the intended purpose is clearly of public benefit, sharing occurs responsibly in a framework of accountability, and the data are securely held.

**Patient and Public Contribution:**

The design of the jury was guided by an Advisory Group including representatives from a health consumer organization. The jurors themselves were selected to be descriptively representative of their communities and with independent facilitation wrote the recommendations.

## 1 BACKGROUND

Every day, large volumes of digitally recorded administrative health data are collected through people's interactions with health systems. Much of this information is held in public sector data collections, such as public hospital and public health insurance records, birth and death registers and notifiable disease registers.^1^, ^2^

Advances in data storage, computing power and analytical techniques are extending the use of digitally recorded administrative health data beyond their historic usage for service development, planning and improvement. Data linkage, in particular, can bring together millions of records from diverse sources to provide both more comprehensive understandings of health and disease states and more accurate identification of rare conditions and responses to treatments.^1^, ^2^, ^3^, ^4^, ^5^ These administrative health data are now routinely shared with researchers working in public institutions, such as universities, under arrangements that aim to promote such research in the public interest while protecting the privacy and other interests of the individual data subjects.^1^, ^3^, ^4^

There is good evidence of public support for sharing public sector administrative health data with researchers, where the data are appropriately protected and the research is in the public interest.^6^, ^7^, ^8^, ^9^, ^10^, ^11^ However, even with protections in place, a number of studies also show that community members remain concerned about data security, the potential for misuse of their data and their lack of control over their own health information.^6^, ^7^, ^8^, ^9^, ^10^, ^11^, ^12^ These studies also show that this disquiet is heightened if private sector organizations have access to the data and that the use of such data for financial gain is an additional significant concern.^6^, ^7^, ^8^

We recently undertook a survey and a scoping review, in collaboration with the Population Health Research Network, that examined community attitudes towards sharing administrative health data with private industry for the development of medications and medical devices.^13^ This work demonstrated broad public support for the secondary use of health data, particularly for health research, even though there were concerns about the risks that attached to sharing such data.^6^, ^7^, ^10^, ^11^, ^14^ Given this potential, there is considerable enthusiasm for sharing government data with private industry^15^, ^16^, ^17^ but also concerns that this could undermine public trust.^6^, ^10^

The findings from this work suggested that both governments and private industry need to address the public's lack of understanding of and trust in the ways in which agencies collect, share, protect and use personal administrative health data. Our national survey found that there was a diversity of opinion in the Australian community and a lack of knowledge about sharing administrative health data, perhaps reflecting variable knowledge and understanding in this domain.^13^ However, these findings reflect the views of participants who may not have had the opportunity to reflect on the challenges found in this complex topic. Put another way, we do not know what informed Australians consider to be justified and legitimate uses of public administrative health data, including uses which require data linkage.

To address this gap, we conducted two citizens' juries in February 2020 to consider the charge:

‘Under what circumstances is it permissible for governments to share health data with private industry for research and development?’

## 2 METHOD

A citizens' jury is a deliberative inclusive method for engaging with the public. Citizens' juries bring together diverse groups of citizens to deliberate about difficult policy questions, often with low community visibility, in a supported, informed and respectful environment.^18^, ^19^ Using a citizens' jury allows a variably informed group of people to rapidly increase their knowledge and understanding of relevant evidence, and social and ethical factors, thereby allowing them to engage effectively with the policy question in an informed manner. A range of methods is used to build the understanding of jurors, including pre‐reading and information booklets, expert presentations and the opportunity for dialogue with experts and each other. The goal of the citizens' jury is to reason together with the common good in mind and to generate policy‐guiding recommendations. This paper reports findings from two deliberative inclusive citizens' juries that considered sharing publicly held administrative health data with private industry for research and development.

For the purpose of this research, ‘Governments’ were defined as Australian governments at the state and federal level; ‘Private industry for research and development’ was defined as companies developing, producing and selling pharmaceutical and medical devices (health insurance and marketing companies were excluded); ‘Research and development’ was defined as including the development, registration, funding, use, and post‐market surveillance of medicines and medical devices in the Australian health system; and ‘Administrative health data’ were defined as data collected in the course of providing and/or paying for a health practitioner and/or health service (eg emergency room visits, prescription receipts, general practitioner visits).

The jury was conducted under the direction of a research team from the Australian Centre for Health Engagement, Evidence and Values (ACHEEV). As much as possible, while recognizing that complete elimination of bias is impossible, the research group attempted to hold a neutral stance on the jury charge.

We formed an Advisory Group to provide guidance for the development of the citizens' juries. The members were chosen to reflect a diverse range of academic and professional backgrounds relevant to the charge, including researchers who use linked health data sets; experts in the legal, social and ethical considerations of linked data sets; organizations providing oversight and facilitation of data linkage research; private industry; and representatives from a consumer organization who advocate to inform, influence and represent the interests of patients, carers and their families.^20^ The Advisory Group members did not sign conflict of interest statements. Efforts to secure a privacy advocate to attend meetings were unsuccessful. However, a member of the research team consulted with the privacy advocate on a number of occasions to ensure we could present the privacy advocate's views appropriately to the juries. The Advisory Group met three times over the duration of the project: twice before the juries to discuss jury structure, content and materials, and once after to discuss the nature and dissemination of the findings. The juries were told that the Advisory Group members were stakeholders in health data linkage and sharing and that the jury recommendations would be provided to the group, relevant organizations and State and Commonwealth Departments of Health. The jurors were told that there was no guarantee that the findings would influence policy but that the work would be published both as a report to ACHEEV and as articles in peer‐reviewed journals. This research was approved by the local university Human Research Ethics Committee (HREC). Our reporting of the work followed the CJ Check framework.^21^

Each jury was convened over 2.5 consecutive days, beginning on a Friday evening, and held in two locations close to Sydney: the satellite cities of Parramatta and Wollongong. This permitted comparison between two different NSW communities: Western Sydney is a burgeoning business, commercial and e‐commerce centre with high‐density living and a relatively youthful, highly educated population (median age 33 years, 34% persons aged 15 years and over tertiary educated) and the Illawarra is a low‐density established industrial hub, historically reliant on coal and steel production, for which the local university is now the largest employer (median age 39 years, 25% persons aged 15 years and over tertiary educated).

Recruitment was conducted by a social research company, Stable Research, from their online opt‐in panel (Stable Soapbox) of more than 110 000 registered panellists. The recruitment strategy was devised to recruit a jury descriptively reflective of Australian population demographics but also to include participants with diverse experience and perspectives from the communities. Stable Research contacted 3043 panel members (2300 in Parramatta and 743 in Wollongong) who met the eligibility criteria, of whom 102 nominated their interest and completed an online screen, with 94 deemed eligible. Participants were selected to fulfil pre‐set criteria to capture a breadth and diversity of backgrounds and experiences with respect to age, gender, postcode, education level and household income (Table 1). For each jury, Stable Research recruited twenty jurors and two ‘standby’ jurors, available in the event of jurors dropping out. Selected jurors were contacted 1 week in advance and again 24 hours prior to commencement of the jury to confirm their attendance. No further exclusion or inclusion criteria were applied. Jurors completed a standard evaluation questionnaire used in previous deliberative work by ACHEEV.^21^ Jurors received an honorarium of $400 and, where appropriate, travel and childcare expenses were reimbursed. In the findings, jurors are identified by a P for Parramatta and W for Wollongong plus a unique identifying number.

**TABLE 1:** Recruitment framework for citizens' jury participants

Gender: 50% female Age: One third from each age range 18–35; 36–55; and 56+ years Employment: diversity across types of employment (eg full‐employment, part‐time, retired, pension, student, apprentice) Household income: 50% below $1486 per week (<$77 272 annual income) based on median Australian income levels Range of postcodes from across the recruitment area Diversity of cultural backgrounds reflecting the local community

Seven days before the jury, jurors received a 44‐page booklet to provide background on the jury process and a balanced overview of the expert witness material, including the potential benefits and risks related to sharing government health data with private industry. The jurors were not required to read the booklet before attending the jury; rather, the booklet supported participants as an additional source of information to increase their understanding of the topic (File S1). An online version of the participant information sheet (PIS) was provided to jurors approximately 2 weeks prior to the jury, with a hardcopy version provided on the Friday night. The PIS described the study aims and premises, funding source and research team's areas of activity. In addition, this information was provided in the Participant Handout Booklet and shared by the research team during the Friday night introductions.

The jury was facilitated by an independent facilitator with extensive experience in community engagement.^22^ The jury convened over dinner on the Friday evening to meet one another, the research team and the facilitator and to develop ground rules. Jurors were provided with information on the jury proceedings and the charge they would consider. In opening the process, the facilitator asked the jurors to reason together on behalf of their communities and with the good of the Australian public in mind.

Both juries followed the same programme over a total of 15 hours on a weekend. Saturday was primarily a day of information sharing and discussion with the experts of the evidence and advocacy views. Members of the research team and advisory group presented information in interactive sessions (Table 2). In addition, the jurors interacted in a world café style exercise^23^ in progressive brief small table sessions with presenters holding the following advocacy positions:

- Health consumers' perspective provided by Health Consumers NSW.
- Private Industry perspective provided by Medicines Australia. This was presented by an industry representative at the Parramatta jury and, due to illness, by members of the research team at the Wollongong jury.
- Privacy advocate perspective provided by Australian Privacy Foundation. This was presented by members of the research team on behalf of the privacy advocate, who was unable to attend the juries.
- Researcher perspective by the Centre for Health Research Illawarra Shoalhaven Population, University of Wollongong.

**TABLE 2:** Information provided to the jury

Presenter	Evidence area
Representative from the Population Health Research Network	What is government health data, how is it collected, linked, analysed and used? How, where, by whom and under what authority is health data linked and shared in Australia?
Research team member drawing on interview research	What is private industry and how are they involved in promoting health? Four case studies of how the private sector is involved in health (new ways to use existing medicines and devices; effective use of medicine and devices; identifying needs for new medicines; and devices and post‐market surveillance).
Legal expert	What are the laws, regulations and policies around using government health data including privacy law and corporate law.
Bioethics expert	What is a risk and what is a benefit? Examples of risks and benefits related to sharing government data with private industry.
Bioethics expert	What are the ethical issues generally and in the specific case of sharing with private industry? Consent including opt‐in/opt‐out/no‐consent mechanisms. National statement.
Research team member drawing on survey research	What does the Australian public think about sharing government health data generally and with private industry?
Research team member drawing on a scoping review	What do international publics think about sharing government health data generally and with private industry?

Throughout, jurors were able to challenge the evidence and views presented.

Sunday was spent in deliberation and development of recommendations. The aim of the deliberative process was to support jurors to critically consider the evidence and the views presented and to deliberate with one another about the implications. Initially, jurors discussed the extent of their support for data sharing and their perceptions of the risks and benefits. The jurors deliberated in small and large groups on the charge and then self‐selected into four groups to discuss and report on focused questions (Table 3). When the jury reconvened, a member of the research team typed up the recommendations from each small group as they presented. These recommendations were then presented back to the jury and jurors revised them together, supported by the facilitator. Finally, the jurors voted on each recommendation. Votes and reasons for and against each recommendation were recorded.

**TABLE 3:** Small group questions

Group	Question
1	Who should have direct access to the data?
	Who should oversee and make decisions about the sharing of data?
2	Do certain types of data need more protection than others?
	Should there be particular limits on the purpose for which the data can be used?
3	Should particular penalties be applied if companies break the rules or misuse the data?
	Who should pay the costs associated with sharing of data?
4	How much should the public be told about the way in which data is collected and shared?
	Should there be a requirement that all results be released?

## 3 FINDINGS

The Parramatta jury had 19 participants drawn from suburbs across western Sydney (initially 20, with one juror dropping out due to illness). The Wollongong jury had 20 participants drawn from across the Illawarra region. Overall, 85% percentage of jurors described their health as good to excellent and over 40% had a university qualification. Table 4 shows the key demographics of each jury.

**TABLE 4:** Key demographics of the participants in each jury

	Parramatta jury ‐19 participants (%)	Wollongong jury ‐ 20 participants (%)
Age (years)		
18‐35	15.8	25.0
36‐54	47.4	40.0
>55	36.8	35.0
Gender		
Male	52.6	50.0
Female	47.4	50.0
Highest educational attainment		
Year 10/year 12	21.1	10.5
Trade/apprentice/TAFE cert	36.8	42.1
University/higher degree	42.1	47.4
Employment		
Full time	68.4	42.1
Part‐time	10.5	10.5
Other (home duties, unable to work, student, retired)	21.1	42.1
Unemployed	0.0	5.3
Relationship status		
Married/de facto	42.1	70.0
Single	47.4	15.0
Other	10.5	0.0
No answer	0.0	15.0
Health status		
My health is poor	0.0	5.0
My health is fair	15.8	10.0
My health is good	36.8	40.0
My health is very good	36.8	20.0
My health is excellent	10.6	25.0
Chronic health condition		
Yes	29.4	35.0
No	64.7	65.0
Not sure/don't know	5.9	0.0
Care for someone with chronic health condition		
Yes	0.0	25.0
No	94.1	65.0
Not sure/don't know	5.9	10.0
My health record^a^		
Yes	55.6	40.0
No	22.2	20.0
Not sure/don't know	22.2	40.0
Worked in health industry and/or in health services or research		
Yes	15.8	15.8
No	78.9	84.2
I prefer not to answer	5.3	0.0
Prescribed medication^b^		
Yes	61.1	70.0
No	38.9	30.0

^a^ Participants were asked if they had a My Health Record. My Health Record is an Australian Government initiative to bring an individual’s health information together. This includes information from the individual, their health‐care providers and Medicare, Australia’s universal health‐care funder.

^b^ Participants were asked if they took prescribed medication(s). A prescription medication (also prescription medicine) is a pharmaceutical drug that legally requires a medical prescription to be dispensed.

### 3.1 Juror recommendations

All jurors, bar one (P4—male, 20 years), in principle supported sharing government administrative health data with private industry for research and development; however, there was a range of perspectives from enthusiastic support to guarded acceptance. In general, the jurors used the terms ‘circumstances’ and ‘conditions’ interchangeably when providing their recommendations. For all jurors, support was conditional and the juries’ recommendations specifying these conditions related closely to the concerns identified in deliberation (Table 5). Despite difference in demographics and setting, the two juries arrived at very similar sets of recommendations completely independent of each other.

**TABLE 5:** Final recommendations from citizens’ juries held February 2020

Parramatta jury	Wollongong jury
**Access: Who should have access to the data?**	
1. We expect the following groups to have access to data: Universities, government departments, pharmaceutical companies, device manufacturers.	1. Only government agencies (one or more) should have direct access to raw data. 2. Private industries should only have access to data once their applications for data have been approved by the independent panel. 3. Private industry should not on‐sell data to third parties.
NB. The jury did not provide a recommendation on individual consent as the jury saw this as unworkable given the nature of the data collection.	Consent: Opinion was divided on this issue. 4a. People should be able to opt out of having their data shared (supported by 7 jurors). 4b. Subject to approval by the independent panel, there should be a waiver of consent for sharing data. (supported by 13 jurors)
**Protection: Do certain types of data need more protection than others?**	
2. All data must be de‐identified – no name, date of birth and address. 3. Sharing data must also comply with other protection laws (eg privacy, child protection, discrimination, elderly people, refugees, etc).	5. Raw data should be very tightly held, protected and not shared. 6. Individual‐level data should only be released for purposes that will contribute to public good. (split decision: Yes 13 No 1 Undec. 6)
**Oversight: Who should oversee and make decisions about the sharing of data?**	
4. An Independent Body for Administering Data Sharing (IBADS) should oversee data sharing to manage applications to access data, linkage and provision of data, auditors, and contractual arrangements. (1 dissenting vote) 5. There should be a tiered system with controls on access, use and disclosure at different levels where IBADS decides on level of access, based on the application, including the company's prior behaviour. (split decision: Yes 14 No 2 Undec. 3)	7. An independent trusted stakeholder panel should oversee and make decisions about sharing data. 8. The independent panel should consist of university researchers, IT and data experts, ethics and privacy experts, private industry representatives, health department representatives, and consumers and community members. 9. The independent panel should be paid, and the payments disclosed, for their contribution to the panel. 10. The application to the independent panel should include, but not be limited to, who will use the data, what data will be needed and from where, for what purpose, how it will be kept secure, how it will be analysed, how long it will be kept, and how and when it will be destroyed.
**Purpose: Should there be particular limits on the purpose for which the data can be used?**	
6. Data can only be used for research and development, not for other uses. 7. A clear, articulate and enforceable definition of ‘research and development’ is needed if private industries are to access data.	11. Data can only be used for the purposes specified in the application to access the data. (2 dissenting votes) 12. Individual‐level data should only be released for purposes that will contribute to public good. (split decision: Yes 13 No 1 Undec. 6)
**Accountability: Should particular penalties be applied if companies break the rules or misuse the data?**	
8. There must be penalties for companies which violate requirements including exclusion from access to data in the future. 9. There must be penalties for government agencies, IBADS, and individuals if they are found to have done something wrong. 10. There should be a well‐resourced ombudsman to investigate complaints about private industry use of government health data.	13. It should be a criminal offence to misuse data. 14. Companies and their directors should be held accountable for any misuse of data in their organization. 15. There should be penalties for companies, including directors, if they misuse data. These penalties should be proportionate to the seriousness of misuse. 16. Misuse of data by private industry should be publicly disclosed.
**Costs: Who should pay the costs associated with sharing of data?**	
11. Private industries should pay for data access.	17. Private industries should pay for the use of data. These payments should cover the cost of managing and accessing the data and excess funds should be reinvested in the health system.
**Transparency: How much should the public be told about the way in which data is collected and shared?**	
12. There should be transparency and openness with the public when data is shared with private industries. This should include information about which companies have access to data, what data is accessed, how long data is held, security arrangements, whether information is passed on to third parties and how (split decision: Yes 14 No 1 Undec. 4).	18. Subject to approval by the independent panel, information about studies conducted using government health data should be publicly available.
**Reporting: Should there be a requirement that all results be released?**	
13. Companies should not be required to share results with the public from using government health data if it impacts on their commercial interests. (1 dissenting vote) 14. Companies should be required to report findings from their research using government health data to IBADS, regardless of outcome. (1 undecided)	19. The results from studies conducted using government health data should be made publicly available if they are of public interest or concern. 20. Companies should be required to share their results with the independent panel, regardless of whether the results are positive or negative.

Many jurors shifted their perspective from complete opposition to recognition of the value of data sharing during the jury, albeit with safeguards. One juror summarized the reasons for this approach:

Yes, we do need to share it, but there needs to be very, very strict guidelines on approvals, on penalties, because I think there will be data breaches, and it is not the everyday people, I think it is the minority, the people who have conditions or whatever, and it could have disastrous effects for those people ‐ certain racial groups, certain community people ‐ so I think we do need to protect them, because they are the ones who are vulnerable anyway. (P1—female, 47 years)

The jurors stated a range of conditions, described below, which needed to be met for data sharing to be acceptable (Table 5). All recommendations were unanimous unless indicated.

#### 3.1.1 Access: who should have access to the data?

Both juries agreed that government agencies should have direct access to administrative health data. However, the approach to access for non‐government agencies differed between Parramatta and Wollongong. The Wollongong jurors indicated that they trusted government to manage their health data responsibly more than they trusted private companies. Raw data (that is, data with identifying information about individuals) were seen as particularly sensitive and the jury wanted only government agencies to have access to these types of data. They believed that governments collected the data and were therefore already accountable for their management. Many jurors believed that since government, unlike private industry, was not commercially driven, health data would be more secure with government agencies than private companies.

The Parramatta jurors did not make this strong distinction between (more trustworthy) government and (less trustworthy) corporations. In contrast, they argued that trust was something that could be earned or lost by any organization.

I'm saying that I don't trust the private sector, but I don't trust the government, and the government has our information. (P6—female, 60 years)

… Concerned about the lack of privacy protections in this country … they are pretty weak here in Australia as compared to other countries. So I am concerned about that, but trust needs to be earned. (P7—male, 37 years)

They favoured formal licensing arrangements for demonstrating and ensuring trustworthiness. Jurors sought to make the concept of trustworthiness more tangible by requiring data recipients to have:

- a stated goal for use of the data;
- general competence in data analytics and in the specific data analysis promised;
- capacity for secure data storage;
- a record of good behaviour with public data sets.

The Parramatta jurors suggested that, provided applicants followed a strict application process, all parties (regardless of type of organization) should have ready access to data. One juror, echoing the views of many, explained:

Yes, they should go and follow the process where they should actually expressly state their goals, their intention of why they are going to use the data. So if that is right in the eyes of the bodies who are actually overseeing this, then they should be allowed to use the data. (P2—male, 33 years)

There were dissenting voices in the Parramatta jury: one juror (P3—male, 40 years) strongly favoured sharing government administrative health data with private industry and felt that some of the protections recommended by the jury were unnecessary. This juror believed that the type of company who could have access should extend beyond pharmaceutical and medical device companies to insurance and marketing companies. In contrast, another Parramatta juror strongly opposed sharing public data with private companies because of the current lack of data security:

[Breaches] should not occur. It is not something that we should be expecting to occur. So the fact that that level of protection and security doesn't currently exist, I would say don't share the data. (P4—male, 20 years)

#### 3.1.2 Protection: do certain types of data need more protection than others?

Both juries recognized that some types of data are more sensitive and vulnerable to misuse and need higher levels of protection. The Wollongong jury indicated that ‘raw data’ fell into this category and stated that only the government agency or agencies should have direct access to raw data. Similarly, one Parramatta juror suggested:

…sensitive information, like date of birth, name and address, needs a different level of protection than data that might be considered less sensitive and therefore all shared data should be de‐identified. (P5—male, 55 years)

Both juries understood that in some cases re‐identification could be possible, and therefore, additional protections would need to be in place. To this end, the Parramatta jury proposed a tiered system of access with a low tier of protection for aggregated data and higher tiers for data that might be re‐identifiable. Researchers wishing to access administrative health data in any of the tiers would have to justify why they needed the data and comply with existing legislative protections such as privacy, anti‐discrimination and child protection provisions and, in addition, any other requirements the oversight body might impose. The jurors wanted to use existing law and regulation where this was available rather than duplicate unnecessarily. Some of the Wollongong jurors envisaged preventing re‐identification by holding the administrative health data in a protected space. One juror explained:

…they don't leave that place with the raw data. They only leave the place with the insight, what they want, the outcome. So they cannot re‐identify the data, they cannot decrypt the data. (W3—male, 23 years)

#### 3.1.3 Oversight: who should oversee and make decisions about the sharing of administrative health data?

Both juries wanted an independent body to make decisions about who should have access, to which data and under what circumstances, and they also wanted this body to have monitoring and auditing powers. One juror summarized the position of the jury as: ‘*We have to trust someone’*. (W1—female, 69 years) Expanding on this, a Parramatta juror commented:

That body is basically there to go from scratch, which is the application process, and up to the end, the results and monitoring of the outcome and everything. (P2—male, 33 years)

Both juries envisaged that the oversight body would be ‘*a cross‐section of the community’* including clinical and data experts, community members and industry representatives with the expertise required to make informed decisions. The original text of the Wollongong jury's recommendation used the term ‘lay people’ but one juror (W10—female, 50 years) requested that this be changed to ‘*consumers and community members*’ as this description gave more explicit direction about the type of people to include on the panel.

Some jurors, in both juries, thought of the oversight body as a vehicle for business transactions through which ‘*potential customers*’ could apply, be assessed and granted access to data. Many jurors also saw the body as a safeguard that would provide the necessary checks and balances to build trust with the community.

The jurors also debated payment for members of the oversight body with reimbursement of costs and a nominal hourly rate to indicate the value placed on their work and encourage individuals on low incomes to participate. One juror (W8—male, 58 years) saw payment of members ‘*like a jury type of arrangement, where you get an allowance*’. A Wollongong juror suggested that payment would reduce corruption:

If you don't pay them or if you ask them to volunteer, you just risk them coming across corruption from people paying them elsewhere. (W2—male, 28 years)

#### 3.1.4 Purpose: should there be particular limits on the purpose for which administrative health data can be used?

Both juries wanted administrative health data to be used only for research and development. The Wollongong jury addressed this issue by requiring companies to undertake a detailed and structured application process. The following exchange in the Wollongong jury illustrates the nature of the requirements:

W4: I think the most important thing is the intent ‐ why you want the data. (Male, 45 years)

Facilitator: But what is acceptable to you in terms of intent?

W9: Development of new. (Female, 60 years)

W4: Something for the public good, medication, a device. (Male, 45 years)

Facilitator: So research, the development of a product that is going to do good for the public.

W2: Or even to help the spread of a product for better use, for the public good. (Male, 28 years)

W9: Yes. The post market one is always good, too, to see how the product worked. (Female, 60 years)

Two Wollongong jurors, although they supported these recommendations in principle, questioned what would happen if additional uses were identified after application. Discussions about amendment processes reassured one of the jurors but this was not included in the recommendation so the final vote count remained the same. Similarly, the Parramatta jury indicated that the data could be ‘*only used for research and development’*. However, the Parramatta jurors thought that the term ‘research and development’ was ill‐defined and could be challenged legally. They therefore called for clear definitions of the term in this context.

#### 3.1.5 Accountability: should particular penalties be applied if companies break the rules or misuse the administrative health data?

Both juries were unanimous in their support for penalties, including ‘*hefty fines [and] exclusion from further data requests*’ (P3—male, 40 years), in order to provide a strong incentive for private industry to behave well. The idea of serious penalties for organizations or individuals who misuse data or allow data breaches was a strong theme running throughout the Parramatta deliberation and it was reflected in the recommendations. Many of the jurors in both juries saw the threat of penalties as a way to address their lack of trust in the ability of private industry to consistently behave in a way which did not cause individual or societal harm. Jurors also recognized that loss of privacy could be devastating to an individual and that penalties may not be sufficient to redress the wrong. One Parramatta juror summarized this issue as follows:

Yes, penalties should be applied but if we are worried that things can go wrong, let's not do it. It is very personal, private data. So before we go into all of these penalties, penalties do not give compensation to the loser, to the one victim. Penalties are just going to give punishment to the data breachers or data losers, but it is a very bad thing for someone who loses their privacy. (P3—male, 40 years)

The Wollongong jury discussed the meaning of the concept ‘to be held accountable’ at length. One juror summarized the views of many when she said:

I think that private industry has to be held accountable if they do the wrong thing with our data. They have to be held accountable. The only way that can happen is that there are regulations and laws put in place so that they are disincentivised, so that if they do the wrong thing it is going to cost them financially, so they won't do the wrong thing, or if they do, they will minimise it. (W11—female, 46 years)

Some Wollongong jurors believed that directors should not be held to account for data breaches or misuse if they had taken all reasonable steps to ensure valid use and data security. Ultimately, the jury decided that accountability did not require that directors should be held responsible for events beyond their control. Instead, accountability was seen as the capacity to justify actions and show that reasonable steps had been taken to protect the data. The Wollongong jury also discussed whether there should be categories to indicate the severity of data misuse or breach of security but they decided that this was something which was beyond their scope. Regardless, full disclosure of misuse by private companies had unanimous support. One juror suggested:

If you do the wrong thing you should face the consequences…then maybe they won’t do it again. (W1—female, 69 years)

Another juror suggested that damage to reputation would be seen as a greater harm and therefore would be more of a deterrent, than being required to pay a fine.

It has been said, but I think the biggest impact is on reputation. A lot of big companies go, you know what, ‘We can handle a $100 000 fine because we are making a million dollars out of it, we don't care’. But the reputation is what will cost them in the long run. I think that's the biggest disincentive you could have. (W4—male, 45 years)

The Parramatta jury also noted that there needed to be penalties in place to deter poor behaviour on the part of government agencies.

#### 3.1.6 Costs: who should pay the costs associated with sharing administrative health data?

Both juries unanimously recommended that companies should pay for access to administrative health data and that the costs should not be borne by the taxpayer. The Parramatta jury envisaged a number of ways to facilitate this including direct up‐front payments for specific data sets, partnerships by subscription or revenue sharing. Payment for data access was a way to ensure that both the data, and access to them, were highly valued. The jurors also discussed a system through which private industry could fund the oversight body as a signal of good corporate citizenship and to support general access to data. Ultimately, however, the detail of how private industry might pay for the data was deemed beyond the scope of the jury. The Wollongong jury went beyond cost recovery with the expectation that ‘*the cost of the data should cover the cost of regulating the industry and the cost of the independent panel, and any excess … should be reinvested’* (W6—female, 35 years). One juror (W7—female, 47 years) wanted the investment to contribute to improvements in the same area from which data were sourced. However, the rest of the jury did not support this condition, with one juror stating that this might lead to research funding being used inefficiently:

If you are too specific, nothing will end up happening, because each panel or each study doesn't get enough funding to make a difference. (W5—male, 24 years)

#### 3.1.7 Transparency: how much should the public be told about the way administrative health data are collected and shared?

Both juries debated what information private industry should be required to share with the public once they had acquired administrative health data. Initially, some Parramatta jurors called for extensive information about the purpose and ways in which administrative health data were used and processed, including mechanisms for de‐identification, the source of the administrative health data, who had access, how long data would be stored, data security, sharing with third parties and risk assessments. Other Parramatta jurors felt that, although the oversight body should receive information, commercial in confidence considerations might limit disclosure to the public. The final recommendation—that there should be transparency and openness with the public when data are shared with private industries—was supported by 14 of the jurors, with one dissenting vote and four abstentions.

The Wollongong jurors initially formulated two conflicting recommendations about how much the public should be told about how data would be collected and shared. After discussion, they unanimously recommended that the oversight body should decide what information private companies should be required to share with the public. As one juror explained:

It goes back to what I said, that the public doesn't need to know about every study done, and it can skew public views because the public isn't educated on why … so pending that, I trust the panel to review that and decide what we do or don't need to know. (W2—male, 28 years)

#### 3.1.8 Reporting: should there be a requirement that all results be released?

Public reporting of the findings from any research using shared administrative health data was also a contentious issue for the both juries. This was due to the potential for adverse effects on the commercial interests of the company. The Parramatta jury recommended that companies should be required to share all findings with the independent oversight body but not with the public (with one dissenting vote). The Wollongong jury unanimously recommended that outcomes of research should always be shared publicly if they were of public interest or concern, with the oversight body responsible for making this decision.

#### 3.1.9 Consent: should consent be required?

The Parramatta jury did not discuss consent in detail and made no recommendations about it. They decided that it was not practical to obtain consent during data collection from very large numbers of people. They were also concerned that allowing people to withhold consent would lead to selection bias, which would undermine the usefulness of any data set.

P8: Just so it is clear in my mind, have we gone back to a general consensus that it won't work if there is an opt‐in [or] opt‐out scenario? Really the only way for the masses of the Australian public is letting an independent body. (Male, 43 years)

P9: Yes. (Female, 49 years)

P10: We have gone back to that? (Male, 43 years)

P9: Yes. (Female, 49 years)

Facilitator: Do we all agree on that?

MEMBERS OF THE JURY: Yes.

In contrast, the Wollongong jury discussed consent models at length. One third of the Wollongong jurors recommended that individuals should be able to opt out of providing data to private industry and two‐thirds were willing to delegate this power to the independent decision‐making panel.

### 3.2 Jury evaluation

The jurors were very satisfied with the jury experience. Across both juries, on a scale of one (‘not at all’) to 10 (‘very much’), they felt their opinions were respected by the group (mean 9.56 ± 0.75); that they were listened to by the facilitator (mean 9.62 ± 1.02); and that the evidence presented was helpful (mean 9.49 ± 0.62). On a scale of one to five (‘strongly disagree’ to ‘strongly agree’), jurors finished the jury feeling: part of the group (mean 4.47 ± 0.76); that the outcome matched their expectations (mean 4.5 ± 0.69); and that participating was worthwhile (mean 4.89 ± 0.39). Most jurors believed that the views of the jury (4.24 ± 0.90) will influence policy.

## 4 DISCUSSION

This deliberative research provided informed and considered community views from two Australian juries on sharing public administrative health data with private industry for research and development, with a focus on the development and evaluation of new and existing medicines and medical devices. One jury specifically wanted research and development defined more precisely. Some demographics of our jurors were similar, in that most were healthy and well educated. Despite differences in demographics and settings, both juries reached similar sets of recommendations. While both juries broadly supported sharing, the strength of this support varied widely. It was also conditional on the resulting outcomes being in the public interest and tightly regulated. Jurors called for a range of strategies such as oversight by an independent body, penalties for breaches and misuse, and requirements for release of information about the private sector's use of health data. Even though jurors were given no guarantee that their deliberations would influence policy, the research team has had a number of opportunities to feed jury findings into the development of policy and legislation. This is a more substantial impact than similar processes often have.^24^

Developments in data sharing are moving faster than current governance structures, ethical guidelines and public policy can accommodate. In some countries, such as Australia, public consultation about these developments has occurred primarily with data custodians, data analysts and direct end users.^22^, ^25^, ^26^, ^27^, ^28^ Even in countries where more extensive research has been undertaken to gauge public or consumer perspectives, with one notable exception^29^ the private sector has not been the only focus of study.^12^, ^30^, ^31^, ^32^, ^33^, ^34^ The research reported in this paper helps to address this gap and provides insight into, firstly, the reasoning of informed community members with respect to sharing government‐held administrative health data with private industry and, secondly, the measures which should govern such data sharing.

Consistent with international systematic reviews,^6^, ^7^, ^8^, ^9^, ^10^ this work indicates that the purpose of sharing data is critical to community acceptance. In addition, in common with previous studies, the jurors in this study identified a number of concerns about sharing data with private industry. These concerns included unease about data security, the potential for misuse and on‐selling of data and the fear that leaked or hacked data could be used to harass, target, discriminate against or embarrass individuals or groups. The jurors sought to address these concerns through regulation, law and policy. Their recommendations are similar to those of participants in other deliberative and qualitative research in the UK,^29^, ^35^ New Zealand,^30^ Europe,^32^ United States^36^ and Canada.^37^

We acknowledge some limitations. First, some of the jurors’ recommendations referenced concepts (such as ‘public good’) that can be interpreted in varied ways. Jurors in both juries used the term ‘public good’ in the ordinary language sense to mean ‘delivering benefit that is widely available’. A detailed account of how the jurors interpreted these terms is beyond the scope of this paper. Second, despite our best efforts to secure certain presenters for one or both juries, we had to replace a small number of presenters with alternates, resulting in some positions being presented by research team members and slight differences between the juries. However, on the latter point the jury findings were still very similar, suggesting that the recommendations transcend differences in information presented.

The findings reported here will help support enhanced ethical and policy guidance for the public agencies which collect, share, analyse and use administrative health data and enhance communication strategies to improve community understanding of the potential value and risks associated with sharing administrative health data. If public‐private health data sharing is to become a feature of the international data landscape, the onus is on governments, regulators and private companies to ensure that the expectations of the community are met to maintain the legitimacy of, and trust in, health data processes and systems.

## CONFLICT OF INTEREST

The authors have no competing interests to declare.

## AUTHORS CONTRIBUTION

Jackie Street^1^ designed and coordinated the study; conducted the study; contributed to the citizens’ juries; analysed data; drafted the manuscript; contributed to revisions; and approved the final manuscript. Belinda Fabrianesi^1^ contributed to the study design; conducted the study; contributed to the citizens’ juries; analysed data; drafted the manuscript; contributed to revisions; and approved the final manuscript. Carolyn Adams^2^, Felicity Flack^3^, Merran Smith^3^ and Serena Joyner^5^ contributed to the study design; contributed to the citizens’ juries; contributed to revisions; and approved the final manuscript. Stacy M. Carter^1^ contributed to the study design; contributed to revisions; and approved the final manuscript. Sean Lybrand^4^ and Anthony Brown^5,1,^ Judy Mullan^6,^ Luise Lago^6^ contributed to the citizens’ juries; contributed to revisions; and approved the final manuscript. Lucy Carolan^1^ contributed to the study design; conducted the study; contributed to the citizens’ juries; contributed to revisions; and approved the final manuscript. Katie Irvine^7^ contributed to the study design; contributed to revisions; and approved the final manuscript. Coralie Wales^8^ contributed to the citizens’ juries; contributed to revisions; and approved the final manuscript. Annette J. Braunack‐Mayer^1^ designed the study; conducted the study; contributed to the citizens’ juries; analysed data; drafted the manuscript; contributed to revisions; and approved the final manuscript.

## Supporting information

File S1

## Data Availability

Data available on request due to privacy/ethical restrictions: The data that support the findings of this study are available on request from the corresponding author. The data are not publicly available due to privacy or ethical restrictions.
