# Supplementary material for: Sharing administrative health data with private industry: A report on two citizens' juries
Source: Health Expect. 2021 May 28;24(4):1337–48. doi: 10.1111/hex.13268 (PMC8369100; doi:10.1111/hex.13268)
Supplement: Supplementary file 1 — File S1 [file HEX-24-1337-s001.pdf]

Sharing Government Data Citizens' Juries: Participant Handout

AUSTRALIAN CENTRE FOR HEALTH ENGAGEMENT EVIDENCE AND VALUES

The Australian Centre for Health Engagement, Evidence and Values (ACHEEV) is the leading centre for high-quality deliberative and values-based research in health and health policy in the Asia-Pacific region.

We make health-related decisions more inclusive and democratic by constructively engaging diverse stakeholders, and more justified and legitimate by explicitly attending to ethics and values. Our methods include community juries, world cafés, discrete choice experiments, Delphi processes, dialogue groups and social research methods.

ACHEEV brings content experts, ethicists, methodologists, policy actors, health professionals, consumers and citizens together around contested or controversial issues in public health and healthcare. sque, enim quis imperdiet aliquam, nisi lorem hendrerit diam, sollicitudin porta nulla nisi in arcu.

https://www.uow.edu.au/the-arts-social-sciences-humanities/research/acheev/

We acknowledge the Aboriginal custodians of the lands where we work, the Dharawal and Darug peoples, and their elders, past, present and future.

UNIVERSITY OF WOLLONGONG AUSTRALIA

Australian Centre for Health Engagement, Evidence & Values

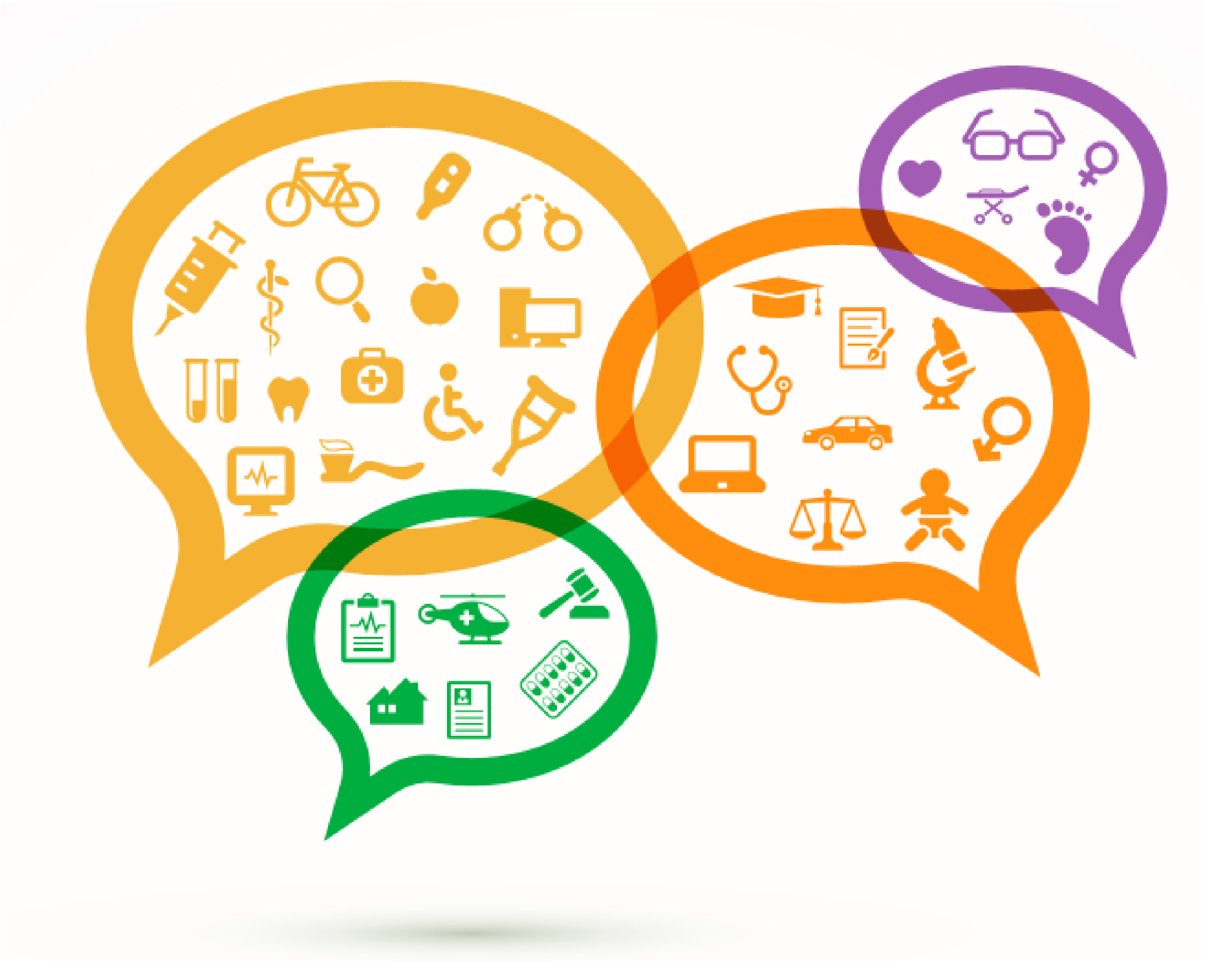

Citizens' Juries:

Sharing Government Health Data

Mercure Hotel, Parramatta, February 7th-9th 2020

Novotel Hotel, Wollongong, February 21st-23rd 2020

This booklet was prepared by a research team from the Australian Centre for Health Engagement Evidence and Values at the University of Wollongong:

Dr Jackie Street
Professor Stacy Carter
Professor Braunack-Mayer
Ms Belinda Fabrianesi

The development of this booklet was supported by the BC Data Deliberation research team including Jack Teng, Michael Burgess, Kimberlyn McGrail, Colene Bentley and Kieran O'Doherty; Carolyn Adams (Macquarie University); Felicity Flack (Population Health Research Network); Katie Irvine (The Centre for Health Record Linkage), and; members of the Sharing Government Data Advisory Group.

UNIVERSITY
OF WOLLONGONG
AUSTRALIA

Australian Centre for
Health Engagement,
Evidence & Values

Table of Contents

SECTION 1: THE CITIZENS' JURY 5

1. What is the purpose of this booklet? 6
2. Why a citizens' jury? 7
3. What is a citizens' jury? 8
4. How do citizens' jury work? 9
5. What happens during a citizens' jury? 10

SECTION 2: SHARING GOVERNMENT DATA 11

6. What do we mean by governments? 12
7. What types of Australian health data are collected? 13
8. What is data linkage? 15
9. Protecting data 16
10. Why might private industry wish to use government health data? 17
11. Are there challenges with data linkage in Australia? 20
12. What benefits and harms might arise from sharing government health data with private industry? 21
13. What are the current laws, regulations and policies? 23
14. What ethical issues do data sharing with private industry raise? 26
15. Consent: Permission options in sharing government health data 28
16. Australian views towards sharing government health data with private industry 32
17. What is happening internationally in this area? 34
18. Summary 36
19. Questions you might want to consider 37
20. Your role in the citizen jury 38
21. What happens to the findings of this jury? 39

Glossary of Terms 40

Images Acknowledgments 43

Notes … 44

SECTION 1: THE CITIZENS' JURY

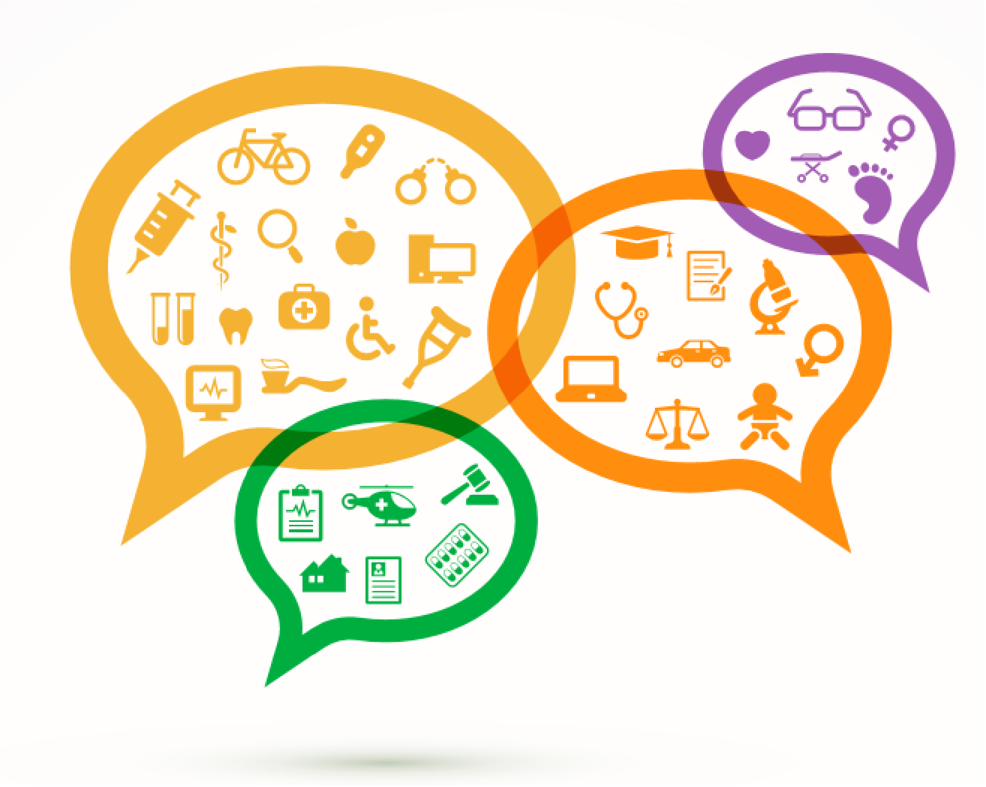

1. What is the purpose of this booklet?

The information in this booklet comes from academic literature, consultations with experts and stakeholders, and recent **research** with the public in Australia and Internationally

The information in this booklet is intended to support you and your fellow participants in the citizens' jury on sharing **government health data** with **private industry** for research and development. It will explain how technology is creating opportunities for research and development by linking groups of data (a **data set**), and how private industry are interested in using government health data for their research and development activities. It will outline some of the potential benefits and potential risks of government sharing health data with private industry. It also describes current Australian **legislation** and **policies** that guide how government health data can be shared for research and development.

You are not expected to be experts on this topic, and you will likely have further questions after reading this booklet. We encourage you to bring these questions to the citizens' jury, along with your insights and perspectives. These are all critical to the **deliberation**.

A glossary can be found at the end of this booklet. Glossary terms appear in bold lettering throughout the booklet.

Please feel free to share this booklet.

We encourage you to bring questions to the citizens' jury as well as any understandings and perspectives you may have

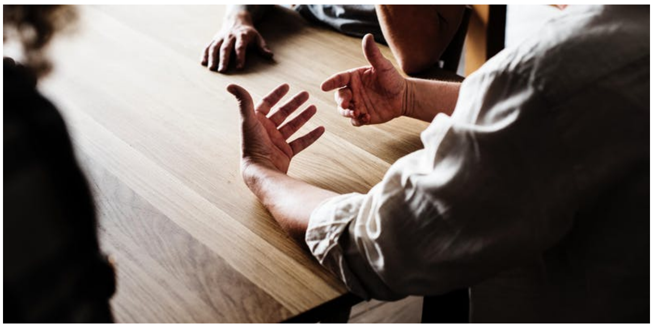

2. Why a citizens' jury?

Key Points:

- A citizens' jury is a community discussion on issues that affect members of the public such as you.
- In this citizens' jury we are asking participants to consider sharing government health data with private industry for research and development.

The citizens' jury you are participating in builds on a recent **research** exploring Australians' perspectives on sharing **government health data** with private industry for research and development.

The **charge** for this jury is:

Under what circumstances is it permissible for governments to share health data with private industry for research and development?

During the citizens' jury you will have the opportunity to interact with experts and people with different perspectives on how data should be stored, shared and used. Over two days we will ask you to consider and deliberate on potential new uses for government health data by **private industry** for research and development, particularly the development, uptake, appropriate use and monitoring of medicines and medical devices.

These new uses and partnerships with private industry have not yet been implemented in Australia. The results of your contributions in this jury may be used to inform the developing **legislation** and **policies**.

In preparation for the event, and over the two days of the event, you will be given written materials to review, hear expert and stakeholder speakers, and explore your perspectives and that of other participants. You will then consider how to balance the diverse interests of all jury members around sharing government health data with private industry.

3. What is a citizens' jury?

A citizens' jury is a community discussion on issues that affect members of the public such as you. People from South West Sydney and the Illawarra regions have been selected to reflect the diversity of life experiences and perspectives in these areas.

A citizens' jury is a democratic process that supports citizens to understand the range of issues and different perspectives associated with a contentious topic. Citizens' juries aim to develop recommendations for government, government agencies, and public and private organisations. They are also useful in identifying areas of concern or disagreement among participants. Policy makers, experts, and stakeholders may provide information or attend the **deliberation** as observers.

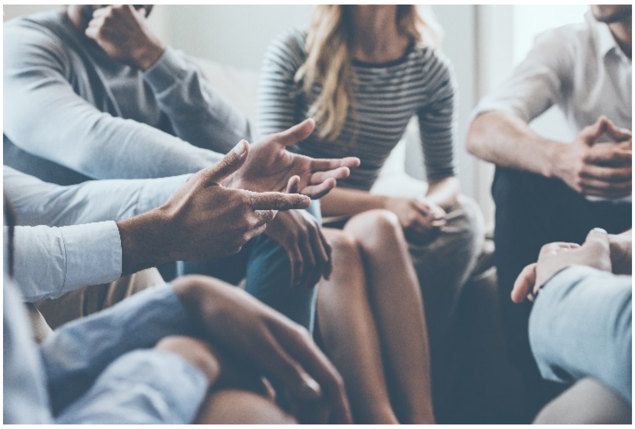

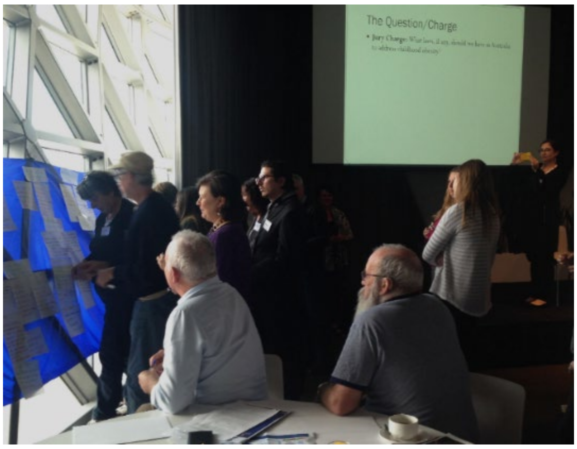

The citizens' jury participants here are involved in an activity to prioritise recommendations developed during deliberation. This jury was held in 2015 and addressed the charge:

What laws, if any, should we have in Australia to address childhood obesity?

4. How do citizens' jury work?

Key Points:

- Citizens' juries invite the public into active participation
- In a citizens' jury participants themselves identify what is important to them about a societal issue and provide advice

Citizens' juries are discussions about important **societal issues** that involve **values** or **trade-offs**. Instead of telling the public how such issues will be resolved, these **deliberations** invite the public into active participation about the issue.

Members of the jury have an opportunity to identify what is important to them about a societal issue and provide advice, in the form of recommendations to policy makers. In this jury, the trade-offs involve balancing the potential benefits of sharing **government health data** with private industry with potential risks of sharing, such as **privacy** breaches.

A citizens' jury is about respecting the diversity of perspectives amongst us and finding ways we can live together.

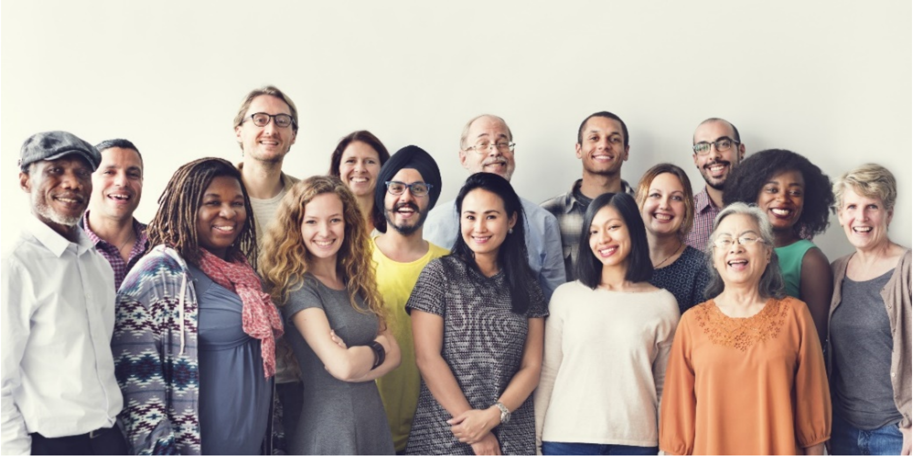

A citizen's jury is about respecting the diversity of perspectives amongst us and finding ways we can live together. The information you read and hear may inform your opinions, and your opinion may (or may not) change. The intent of the jury structure is to inform and support participants as they discuss issues and make recommendations.

5. What happens during a citizens' jury?

Key Points:

- During this citizens' jury you will hear different perspectives from different speakers about sharing government health data with private industry
- You will participate in small group and large group discussions to answer different questions about these perspectives
- Along with your fellow participants you will formulate policy recommendations

On the morning of the first full day (Saturday), you will hear information from experts on different aspects of using **government health data** for research and development by **private industry**. You will also hear from people with different perspectives on how data should be stored, shared and used. These people are chosen to provide a wide range of perspectives and will not necessarily agree with each other. In small groups, you will have the opportunity to ask questions and get more information.

The following day (Sunday) will begin with a brief recap session, and then you and the other participants will work with a trained **facilitator** to identify areas of agreement and difference and attempt to develop a consensus on the jury charge. Based on this, the group will formulate policy recommendations. While the recommendations are important because they represent agreement, there may be times when you and your fellow participants do not all agree. In all cases, we are interested in the reasons behind your perspectives, as this is also important information for policy-makers, particularly where there is no consensus. On Sunday afternoon, participants will have an opportunity to review all of the recommendations together and make any necessary modifications, and then to present those recommendations to the research team and stakeholders.

Example question the jury might consider

Do you think that there may be particular harm to the public from sharing health data?

SECTION 2: SHARING GOVERNMENT DATA

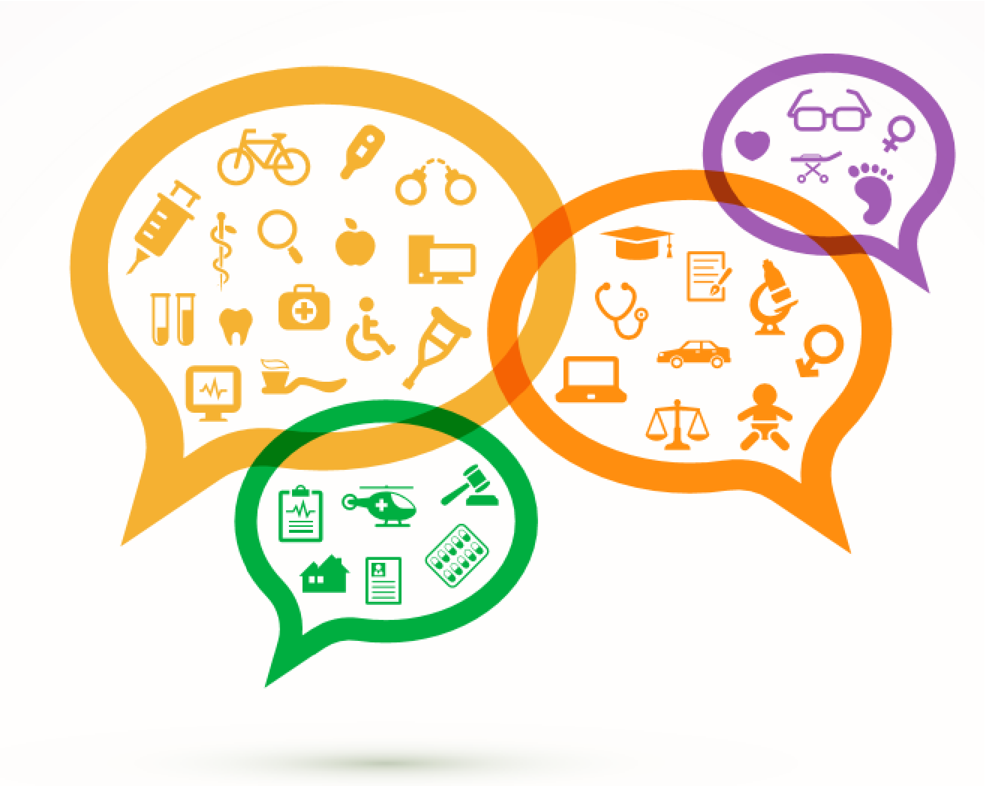

6. What do we mean by governments?

Australia does not have one unified health system; rather we have various connected health systems.

The federal government, state and territory governments and local governments share responsibility for the operation, management and funding of Australia's health system. The main roles of each level of government in Australia's health system include:

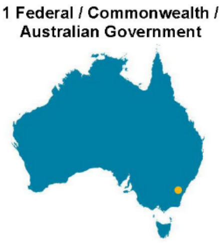

Federal government is responsible for national health policy, administering Medicare (including funding GP and specialist medical services), providing funds to states and territories for public hospital services, oversight of Primary Health Networks, funding medicines through the **Pharmaceutical Benefits Scheme**, regulating private health insurance, funding Aboriginal and Torres Strait Islander health care and funding health and medical research.

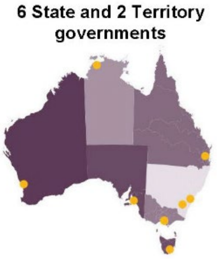

State & territory governments are responsible for funding and managing public hospitals, regulating and licensing private hospitals, providing oversight of local health networks, delivering public community-based and primary health services, delivering preventive services such as cancer screening and immunisation programs, ambulance services and health complaints services.

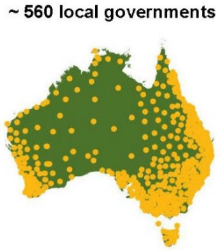

Local governments are responsible for environmental health-related services such as waste disposal, health promoting environments such as green spaces and bike paths, community and home-based health and support services, and delivery of health promotion activities.

During this citizens' jury we are interested in what you think about all levels of government sharing health data with private industry

7. What types of Australian health data are collected?

Key Points:

- In Australia, health data is collected by individuals and organisations who work in the public domain
- These include public institutions such as Medicare, researchers at universities and private industry.

In Australia, health data is collected by both individuals and organizations, such as public institutions, researchers, **private industry** and **private health providers**.

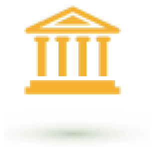

Public institutions include federal (e.g., Medicare) and state organizations (e.g., NSW Ambulance, public hospitals, NSW Health Pathology). Public institutions collect a wide variety of **personal information** including **demographics**, prescriptions, emergency room visits and medical test results.

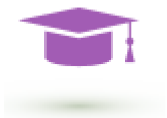

Researchers collect a wide range of data including clinical and survey data. Researchers often work at universities, health authorities, **registries** and public institutions (e.g. Australian Institute of Health and Welfare).

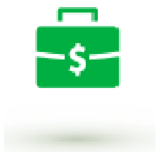

Private industry includes **pharmaceutical** companies (e.g. Johnson and Johnson Medical) and device manufacturers (e.g. Amgen). Private industry collects data through clinical trials of their products which are designed to test whether the products are effective and safe.

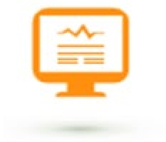

Private health providers, including GPs, medical specialists, physiotherapists, **medical imaging** laboratories, dentists, private hospitals and private pathology laboratories collect data in the provision of services used by the public. Some of this data is also held in government institutions but some are only held by the health provider.

The health data collected by the Australian Government can be categorized into the following types:

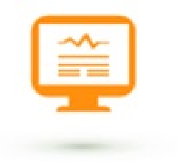

Health **administrative data**: health data collected in the course of providing and/ or paying for health services (e.g. **demographic data**, emergency room visits, hospital admissions, prescription receipts, and Medicare claims).

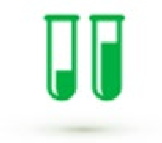

Clinical data: data that includes specific information about persons, their health conditions and/or care (e.g. blood pressure, lab results, **medical imaging**, diagnoses, clinical notes (**electronic health records**), and **genomic data** information.

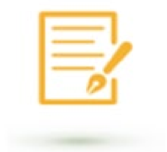

Data from research participation: data collected directly from and about individuals or groups, often through surveys, which may be used for research (e.g. age, ethnicity, income, daily activities, and opinions).

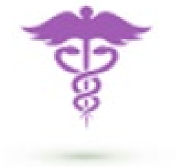

Registry data: clinical data about people with specific conditions (e.g., cancer, diabetes and joint replacement registries).

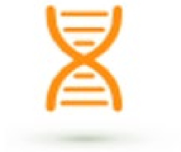

Physical and biological data: data about specific aspects of human biology (e.g., height, weight, blood pressure, blood or tissue samples). Blood and tissue samples collected in a central location are called "**biobanks**."

Private industries also hold health information about people (e.g., about people who have been in their clinical trials) that could be linked with government health data.

Recent advances in information technology mean that it is now possible to handle, link and analyse much larger volumes of data. This change means the volume and types of data being produced and collected by governments has increased greatly.

8. What is data linkage?

Most government health data is stored on **electronic data bases** and can be understood as a **data set,** for example, all emergency room visits in a hospital. While data sets can be used alone for research, researchers can also use them with data linkage.

Data linkage involves bringing together information about the same person from two or more data sets, from two or more different sources (e.g. from an Education Department and a Health Department). The data linkage technical process is complex and designed to minimise risks to **privacy.** One of the ways to reduce risks to privacy is to separate **identifiers** (*name, address, date of birth*) from health information (*for example, reason for hospital admission*) and to separate the roles of the people involved in the process.

Data linkage technical process

The **data linkage unit** receives demographic information (*including names, addresses, date of birth*) from all participating **data custodians**. The same information appearing in the different databases is then joined or linked by matching names, addresses, gender and date of birth.

Are there other ways to obtain the same information as data linkage?

New studies would need to be set up to collect similar information to that obtained by data linkage. This process would be much more expensive than data linkage as all the information would have to be collected anew from many people. The studies would take longer to perform, particularly if the researchers were looking at changes or events occurring over a number of years. The study sample would also not be completely representative of the population as a whole.

Watch data linkage explained by SA-NT DataLink:
https://www.youtube.com/watch?v=vLYGcbxrIPA&feature=youtu.be

9. Protecting data

Key Points:

- **Linked data** used for research is anonymised to reduce the possibility that it could be traced back to a specific individual
- **Anonymisation** includes removing or **encrypting** identifiers such as name, address and hospital record number and reducing the amount of detail in the data.

When data is first collected a person is often fully identifiable. As information which identifies a person is removed (e.g. name or address) it become harder (but often not impossible) to identify a person by looking at the data. Think about a photo of a person as shown here. As we blur the image it becomes harder and harder to work out who that person is. However, if I told you that the image can be found on the website of a particular university with a little searching you would be able to work out who it is.

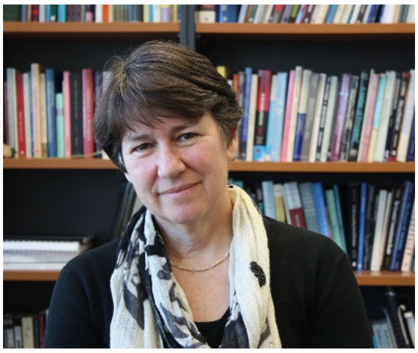

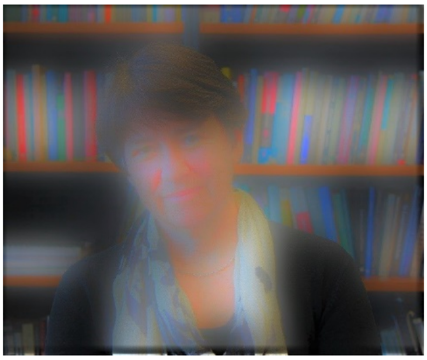

The process of removing information about an individual from a data set is called anonymisation (sometimes called de-identification). This may involve removing **identifiers** such as name and address and reducing the amount of detail in the data. The purpose is to minimise the risks that individuals will be re-identified and have their **privacy** breached. Combining and summarising information into what is called **aggregated data** is also a way to anonymise it. The more detail that is removed from the data the lower the risk of re-identification but the data may become less useful for research.

Researchers are only provided the data that is necessary to conduct their approved research. Anonymisation is not the only safeguard used to minimise privacy risks. There are also legal and contractual obligations and strict data security requirements. Research use of linked data also needs to comply with ethical standards for research.

10. Why might private industry wish to use government health data?

Key Points:

- Government health data is wide ranging and could be easily shared with private industry
- Linking data sets can lead to new discoveries and shed light on complex issues
- Linking government health data could be useful in monitoring the safety, quality and effectiveness of drugs and medical devices. It may also prove useful for decision making by regulators and funders of the health system.
- Linking data sets can also help researchers and policy makers to better understand the characteristics and needs of the population

When we talk about **private industry** involved in research and development in health we are primarily referring to companies producing medicines (e.g. Johnson and Johnson Medical) and device manufacturers (e.g. Amgen). Private industry also includes insurance and marketing companies, but here we will not be considering these sectors.

Government health data sets are large and wide ranging. Because it is **digital information,** the data can be easily accessed, moved, shared, and used. This means that data research can potentially be very responsive: the research can be conducted rapidly and findings implemented quickly. This could be particularly useful for private industries who wish to develop new treatments quickly or monitor their effects in real time.

All of these new opportunities can lead to insights that may be valuable for private industry. Sometimes these opportunities could benefit society but this might not always be the case.

In this jury we will not be considering the use of government health data by private companies for the purposes of marketing or insurance

Here we describe examples of areas where data linkage has been used or might have been used to improve health which, for a range of reasons, also may be seen to be of value to **private industry**:

The development of new ways of using existing medicines and devices:

Off-label use of medications is common. The Council of Australian Therapeutic Advisory Groups has advised that off-label use should only be considered "when all other options, including the use of medicines approved by the **Therapeutic Goods Administration**, are unavailable, exhausted, not tolerated or unsuitable". Understanding off-label use may be helpful to warn doctors about potential risks in the use of particular medicines in this way and also may be useful for private industry to understand the potential for expanded regulated use of their products. For example, if off-label use of a medicine is high the company may consider undertaking the necessary research and submission procedures to support the use of the medicine within the **Pharmaceutical Benefits Scheme**.

Effective implementation of evidence-based recommendations for the use of medicines and devices:

Private industry are interested in the way in which new medicines and medical devices are used in health care in Australia. It is in the **public interest** (and the company's interest) to make sure that a new treatment that works better than old treatments is used as widely as possible. Using government health data can demonstrate how well new treatments work and could be used to reduce side effects or complications and therefore costs.

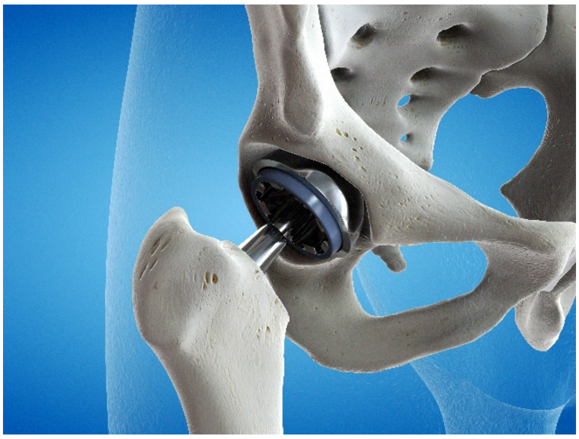

Assessing the need for new medicines or devices:

Understanding the extent of need for treatments for a particular disease or disorder can be useful for private industries in their decision making. For example, **government health data**, including linked data, may be useful for a company to establish a business case for the development of a new medicine or device or may support a submission for the inclusion of a medicine on the **Pharmaceutical Benefits Scheme** (PBS). In 2016 the Australian Department of health released a **de-identified** data set containing 10% of **Medicare Benefits Schedule (MBS)** and Pharmaceutical Benefits Scheme (PBS) data. This gave many private companies a useful tool. For example, it provided insight into the use of alternatives to particular devices which companies may have been thinking about bringing to the Australian market. However, without the appropriate protections in place, it also laid the data open to re-identification and the risk of **privacy** harms.

Post market surveillance:

Post market surveillance is the practice of monitoring the safety of a **pharmaceutical** medicine or medical device after it has been released on the **market**. A private company could conduct post market surveillance on one of their medicines by linking the Pharmaceutical Benefits Scheme (PBS) claims data set with government hospital admission data to provide insights on side effects. It is more difficult to monitor the use of devices since the Medical Benefits Schedule (MBS) documents services not the device used in that service. A device may be monitored through a **registry** such as the Australian Orthopaedic Association National Joint Replacement Registry. Currently not all devices are covered by a registry.

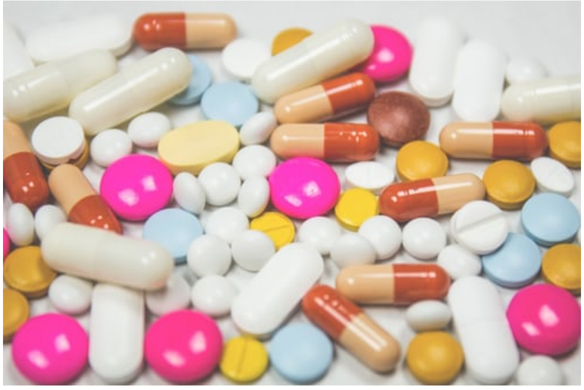

11. Are there challenges with data linkage in Australia?

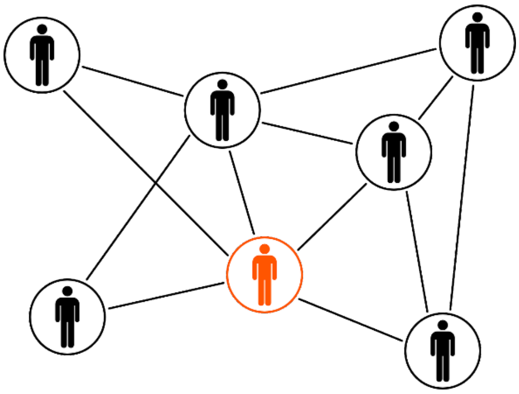

The data linkage process can be time-consuming, partly because Federal and State laws with different requirements may need to be considered.

Other challenges in gaining access to database information for research include:

- Identifying the right **data sets** for the research purpose
- Identifying the right **data custodian**
- Getting permission from the data custodian
- Getting ethics approval
- Getting the correct data
- Checking the data for accuracy and missing or incomplete information

Often research using linked data sets requires permission from multiple ethics committees and multiple data custodians with different requirements for access to the data sets.

12. What benefits and harms might arise from sharing government health data with private industry?

Key Points:

- Decisions about sharing government data with private industry involve balancing the benefits and the harms that may occur.
- Benefits include better treatments or prevention of disease.
- Harms include an increased risk of data breaches (release of data outside the intended use).

Deciding when it is okay for **private industry** to use **government health data** involves balancing the benefits (or 'goods') against the harms (or 'bads') that may occur. With both benefits and harms, we need to think about the likelihood that the benefit or harm will arise and how big that benefit or harm would be. When we talk about the likelihood of harms arising, we describe these as risks. There is a long list of possible benefits and harms, and here we will only mention a few.

Benefits

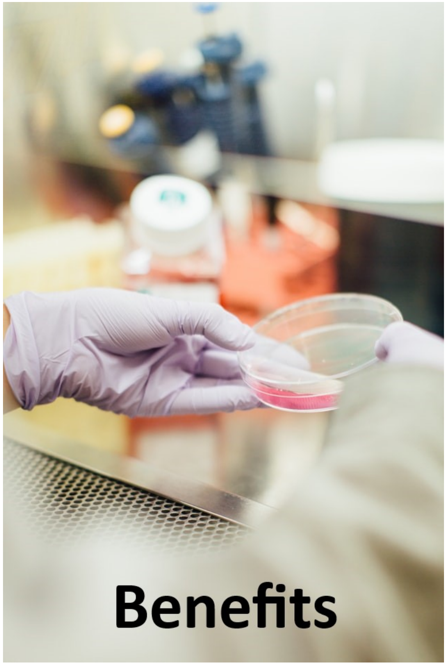

The benefits that may arise if private industry has access to government health data begin with better prevention or treatment of diseases or better health services for the people who need them now. There are also potential benefits for other people, both from the specific treatment that would be available to them in the future if they needed it and from other new treatments that may be developed as a result. Then there are benefits such as a flourishing biomedical industry which provides satisfying and secure employment. Many people also describe better scientific understanding and knowledge as a benefit in its own right.

Harms

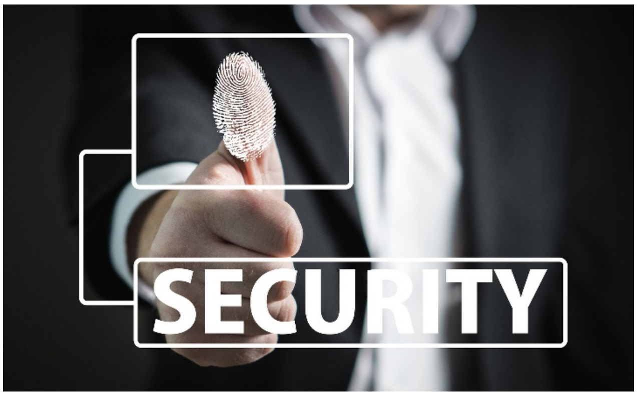

On the other side, there is a risk of harms arising from giving **private industry** access to government health data. For example, there is a possibility that data may be released to people who should not have access to it (a **data breach**). This could be harmful for the individuals whose data is released, and it could also be harmful for society more generally. Inappropriate release of our health data could undermine our trust in our health system so that people are less likely to use health services when they need them.

Who benefits or is harmed?

It is also important to think about who could benefit from or be harmed by giving private industry access to **government health data**. Some people want to be make sure that benefits from sharing the whole population's data will extend beyond just those people affected with a specific disease, particularly if that disease is a rare one. Others are concerned about sharing government health data with private industry if the main (or only) beneficiary will be a company's directors and shareholders.

13. What are the current laws, regulations and policies?

Key Points:

- Personal health information is highly regulated
- Each Federal, state and territory government has its own laws and policies.

An individual's health information is personal and sensitive and is, therefore, generally collected on a confidential basis. Such information is protected by a range of laws and policies that aim to ensure that the information is only used in ways that people expect and not for other unrelated purposes. Personal health information can only be shared by these agencies in certain specific circumstances, for example, where you have given your **consent** or where sharing the information is otherwise allowed under **legislation** or **policy**. These laws and policies aim to balance the benefit to the community with protecting the **privacy** of individuals.

Each federal, state and territory government has its own laws and policies in relation to the collection, use and sharing of this information including public health and privacy laws and policies.

In the next section we have focused on examples from the federal level to illustrate how these laws and policies work in practice.

Public health laws and policies

The federal, state and territory governments all have legislation that regulates the use and sharing of health information collected under the legislation. At the federal level, for example, the *National Health Act 1953* regulates the **Pharmaceutical Benefits Scheme** (PBS). The Act requires that, whenever a medicine on the PBS is dispensed, the pharmacy must provide the Australian Government with information about the identity of the individual, the prescribing doctor, the prescription dispensed and the supplying pharmacy. It

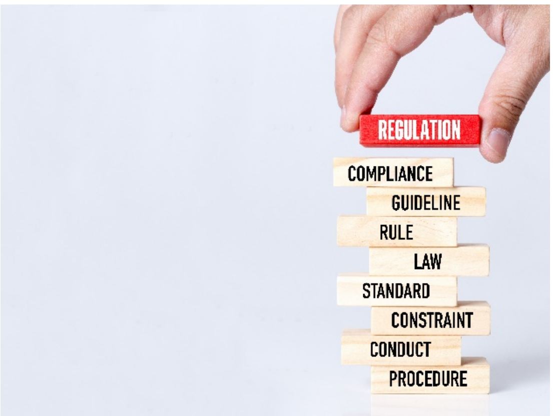

is a **criminal offence** to disclose this information except in specific circumstances.

The Australian Government Department of Health *Data Access and Release Policy* makes clear that this information is a valuable community resource and should be made available for research, while respecting **privacy** and managing risks. Therefore, the *National Health Act* allows the information to be shared in certain circumstances. For example, the Australian Government may publish statistics based on the PBS data so long as the information is published in a way that does not identify a particular person.

In some circumstances, however, agencies may want access to identifiable **personal information**. In order to release this kind of information, the Minister for Health must certify, in writing, that it is necessary in the **public interest** (for example, to conduct research that will benefit the public). Public sector agencies must always act in the public interest. Private sector companies, however, are required by law to act in the interests of their **shareholders**, rather than in the public interest more generally. This makes it difficult to certify that research undertaken by, for example, a **pharmaceutical** company would be in the public interest.

Human Research Ethics Committees

Where a research project uses **individual level data**, it must be approved by a **Human Research Ethics Committee** (HREC). HRECs are required to decide a number of things, including whether:

- the research requires access to individual level data;
- it is impracticable to seek consent from the individuals involved;
- the research is in the public interest;
- the public interest in the research outweighs the public interest in preserving the privacy of the information.

Privacy laws and policies

As well as public health legislation, there is privacy legislation (laws and rules) at the federal level as well as in most states and

territories in Australia. Privacy legislation regulates the collection, use and sharing of **personal information** more broadly, including health information. In some jurisdictions, including NSW, there is also specific health **privacy** legislation.

The federal *Privacy Act 1988* covers the federal public sector and the private sector. State and territory privacy legislation generally applies to state and territory public sector agencies. Despite this patchwork of legislation and policy, each is based on similar principles and contains similar requirements. While public health legislation applies specifically to personal information collected under that legislation, privacy legislation applies more broadly to all personal information collected by government agencies.

Privacy legislation and policies allow personal information to be used in some circumstances without individual **consent** where the use benefits the community. Such research must be approved by a **Human Research Ethics Committee**.

14. What ethical issues do data sharing with private industry raise?

Key Points:

- Ethics in data sharing involves respecting people's autonomy, people's right to privacy and whether to use available data to benefit society
- Ethics in data sharing is about balancing individual rights with benefits to the whole community.
- Individual rights protect us against intrusions, allow us to live autonomously and enable diverse societies to exist together harmoniously.
- Communities cannot function if individual rights are always considered more important than any other values and people do not give up some rights for the common good.

To better understand the ethical issues with sharing government health data with private industry, consider the following questions:

- Should my personal health information be used only for me or should it also be used to help others in my community?
- What are the important ethical principles that we could use to argue for or against sharing **government health data** with **private industry**
- How do ethical theories help us deal with the ethical question: Is consent required for sharing with private industry?
- If consent, or permission, is required, what kind of consent is better?

Ethically important principles in sharing health data

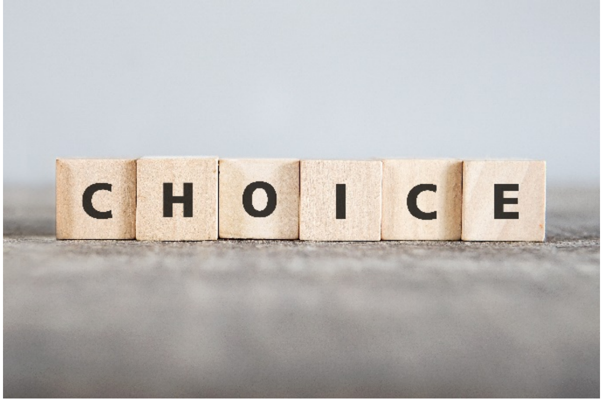

Respecting people's autonomy: People have the right to make choices about their lives. Consent helps people express their choices. Other values sometimes override respect for **autonomy**.

People's right to privacy: Part of **privacy** is controlling access to information about ourselves. Everyone should respect our privacy. Other values sometimes override privacy rights.

Using available data to benefit society: It may be unethical to be in a position of being able to benefit people, but to then not do it.

Ethical theories

Utilitarianism: Promotes the greatest good for the greatest number of people. It examines consequences that would arise from an action or inaction and does not give preference to any one person or group.

Right-based theories: Promotes the idea that people should be able to live **autonomously** and that is essential to respect people's rights, e.g. **privacy**

Communitarianism: This values the benefits and protections provided by organised society. It promotes the idea that people should contribute to the community and it may be necessary to 'sacrifice' some rights, e.g. privacy.

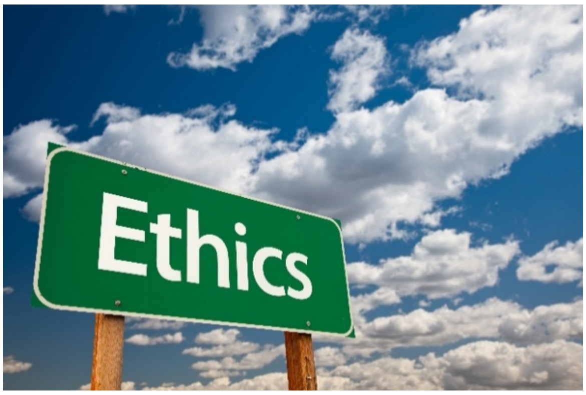

Individual rights vs. community benefit

Individual rights: Protect us against intrusions. Allow us to live autonomously. Enable diverse societies to exist together harmoniously

BUT

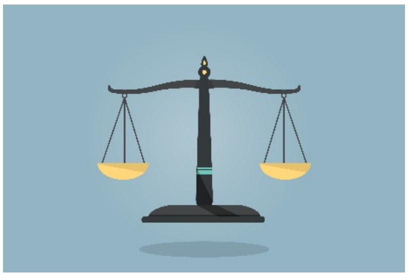

Communities cannot function if: Individual rights are always considered more important than any other values. People do not give up some rights for the common good

15. Consent: Permission options in sharing government health data

Key Points:

- There are three options for consent: opt-in consent, opt out consent and no consent. Alternatively the data linkage may be considered unacceptable.
- In Australia, data linkage projects can go ahead without consent from the people the data relate to providing there is approval from a Human Ethics Research Committee.
- Even with ethics approval, there may be other barriers including: shortage of funds and time required to contact participants.

There are four options for data linkage and obtaining **consent**:

1. **data linkage** is not acceptable in any circumstances;
2. opt-in consent**;**
3. opt-out consent;
4. no need to ask for consent.

These options are relevant to sharing **government health data** with any individual or group, and not just with **private industry**.

Data linkage is not acceptable

Some people may feel that when they provide health information for treatment purposes, this information should only be used for that purpose, or directly related ones, but not any other activity, such as research, even if benefits result from the research.

What is opt-in consent?

Opt-in consent requires express or active consent and means that people or their information are included only if they consent. When people opt-in, they are usually asked to sign a consent form or, in some cases, to give their verbal approval.

What is opt-out consent?

Opt-out consent means that people or their information are included unless they expressly request not to be included.

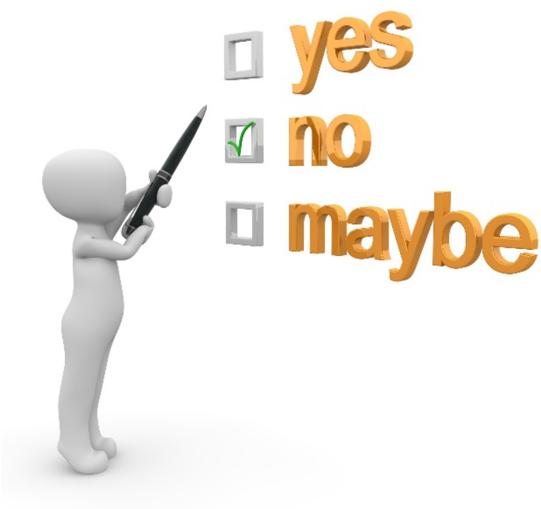

Although opt-out consent is not widely used in Australian research, it can be an effective way to achieve consent for some types of research or health care programmes. One example where opt-out consent would be useful would be a stroke registry where people's information would be included at the time of treatment and retained unless they later asked for it to be excluded.

What does no consent mean?

Even though **consent** is important, most of us recognize that our personal preferences cannot always be taken into account, especially for activities affecting the whole population. For example, public health policies about infectious diseases do not require consent for collection and use of information because any level of consent refusal could lead to the failure to contain spread of such diseases in the community. In the same way, including everyone in a particular database would increase the value of a **data linkage** project.

In Australia, research with government health data can go ahead without express consent if a **Human Research Ethics Committee** reviews and approves the research.

Consent in research with government health data

Many researchers think that having to ask for consent to use people's health data makes the research impractical to do. This is because of:

- the cost and time needed to contact thousands of potential participants

- the difficulty in locating individuals due to changes in their circumstances e.g. moving, or deaths since the records were first created;
- the fact that people who do consent share different characteristics to those who do not consent. This creates bias in the research findings, making them less reliable.

Evidence from a South Australian study of different ways people preferred to consent.

A total of 1,129 families were recruited in a South Australian study examining different ways of consenting for linkage of their baby's hospital information and vaccination history for monitoring vaccine safety:

- half were randomly allocated to the opt-in process and,
- half were randomly allocated to the opt-out process

There were 925 (82%) parents involved in a follow-up interview.

1. Very few parents opted in (21%). On the other hand, few parents opted out and participation in this group was high (96%).
2. The people who opted in were more likely to be married, older and better educated than those in the rest of the study. The people who did not opt out were similar to everyone else.
3. Around 25% of parents either didn't actually do what they said they wanted to do about opting in or out, or misunderstood what they were being asked to do.
4. Most parents indicated they wanted to be asked for consent for data linkage in one form or another;
    - 42% preferred opt-out consent;
    - 24% preferred opt-in consent;
    - About 30% indicated that consent was not necessary.

Very few parents opted in (21%): participation was low.

On the other hand, very few parents opted out so participation in this group was high (96%).

The study showed that opt in consent was not an effective way to conduct research that represented the whole population.

16. Australian views towards sharing government health data with private industry

In June 2019 we conducted an online survey, looking at attitudes towards sharing government health data with private industry for research and development, with 2,537 members of the Australian public.

What do the Australian public think about sharing health data with private industry?

Our survey showed that the Australian public:

- Are uncertain about sharing with private industry
- Prefer opt-in method of consent
- Show strong support for a number of conditions to be met before their health data is shared
- Do not trust private companies and regulation
- Do not trust the Australian governments' infrastructure and staff to share in a safe and ethical way.

We found that Australian participants were uncertain about the Government sharing their **anonymised** (de-identified) **government health data** with **private industry**, with only 52%-58% of all participants supporting private industry use of health data for research, development of new drugs or devices, and to improve health services.

When asked about their preferred method of **consent** 55% of participants requested opt-in consent (It is worth noting that it would probably be impossible to get consent from all individuals in the **data set**).

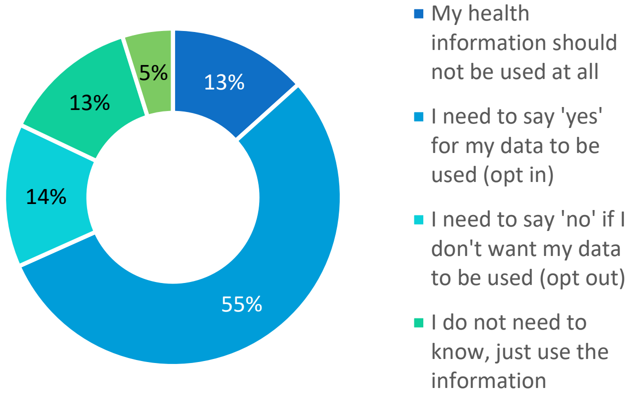

Participants were given a scenario in which the government had decided to share their health data with a private company and they were invited to indicate how important various conditions would be for sharing. Participants showed strong support for:

- Strict **regulation** of data storage and sharing;
- Research for **public benefit**;
- Oversight by an independent **ethics committee**;
- Release of all research findings;
- **Transparency** in data sharing, and;

- Payment by commercial bodies for any data.

We gave participants some statements to find out their views about **private industry** and their use of health data. In general, most people did not trust private industy or governments with their data.

Overall, women, younger people, less well-educated people, people living in regional areas and, to some degree, people with poorer health status, were more concerned to put conditions on the release of **government health data**.

Additional comments in the survey highlighted a general lack of trust in private companies as well as the Australian Government. Participants were reluctant to share health information with private companies if the end goal is profit generation and not societal benefit. One participant wrote: *... private companies are only in it for self-interest and not for the good of humanity because the mighty dollar drives them and nothing else*. In addition, the participants referenced the poor track record of the Australian Government in handling data and they questioned the ability of Government to keep their data secure and prevent misuse. One participant wrote: *I am also not sure whether the government can be trusted to use and manipulate my health data properly and just give the external company only the information they require*. There also appeared to be difficulty in understanding the nature and purpose of research using linked **data sets**, with one participant writing: *I don't think that that information is any use to anybody for developing new drugs or procedures.*

The research also highlighted the need for increased **consultation** with an informed public, such as participants of a citizens' jury, to inform how trust in the Government and private industry, around data sharing, could be built with the Australian public.

17. What is happening internationally in this area?

In April 2019 we conducted a review of studies from the last ten years which looked at community views in Australia and other countries on sharing of government health data with **private industry**. We found thirty three papers that reported these views. Twenty four of these were from the UK and USA. There were no studies in which Australians had been asked for their views.

What do the public (internationally) understand about data research?

Our review showed that the general public in other countries do not understand:

- How the health sector works in their country
- The value and nature of research which uses data
- How much and where health data is being collected and shared
- The ways in which different organisations and departments work separately and together to conduct health research
- The safeguards that are in place to protect data sharing and use

People internationally were concerned about a range of issues including that the data would not be used for **public benefit**. People were worried that data might be used for the benefit of private industry rather than the public. They were also concerned that data might be used for purposes that were harmful to individuals or groups. They were concerned that data might be sold on, hacked or released and that this would have an impact on people’s employment, insurance, health care and financial services.

Some people were concerned that private industry would use the public data to make a profit for their shareholders and then sell the outcomes of their research back to their government. They felt that private industry was less accountable to the public and were less likely to hold the **public interest** or work for public benefit.

Despite these concerns, many people valued the benefits of sharing **government health data** with private companies and were willing to share their data provided a number of conditions were met. These included:

- The research should be of public benefit and in the public interest
- The data should be securely stored
- Access to the data should be tightly controlled
- The data should be **anonymised**
- There should be independent oversight of how data is shared and used
- There should sanctions (e.g. fines) for misuse of data

Consent was a highly contested issue. Some people wanted to be able to consent every time their data was used while others either did not see the need for any consent or were willing to support data sharing without consent if there was high **public benefit**.

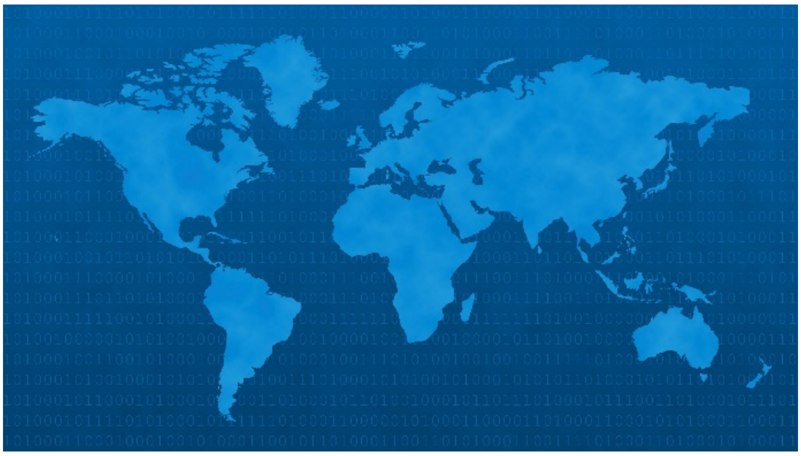

18. Summary

New and more data is being collected about us every day. New sources of data can be used to create new **data sets** in new contexts and with new partnerships.

One of these new partnerships is between governments, which hold large health data sets, and **private industry**, which make new drugs and health care devices. Many people believe there are particular challenges in sharing government health data with private industry.

The same things that make new data-based research opportunities so promising and powerful also raise numerous issues including maintaining **privacy,** protecting personal **autonomy**, preventing harms and promotion of the ethical use of data. It is therefore important to develop **policies** and **regulations** that can balance the benefits to society with the potential risks of sharing the health data held by governments with private industry for research and development.

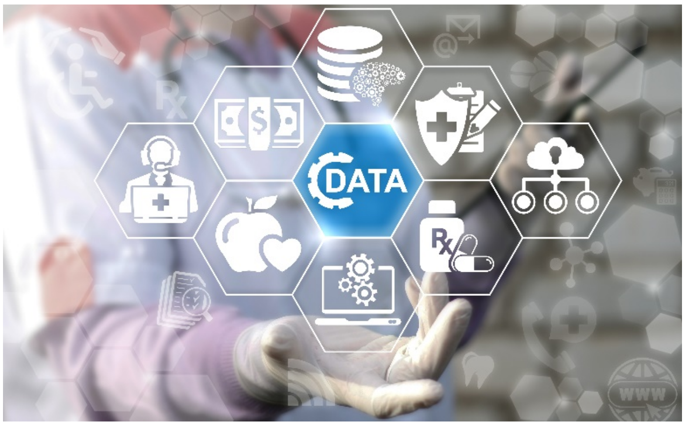

19. Questions you might want to consider

We are asking you about how you think our society should balance the risks and benefits of research using linked government health data.

In doing so you might wish to consider the following questions:

1. What benefits and harms do you think might occur due to sharing data with **private industry**?
2. Assuming governments decided to share data what rules or conditions should be in place to regulate the ways in which the data is used and stored:
   - Who should have direct access to the data?
   - Who should oversee and make decisions about the sharing of data?
   - Do certain types of data need more protection than others?
   - Should there be particular limits on the purpose for which the data can be used?
   - Should there be particular penalties applied if companies break the rules or misuse the data?
   - Who should pay the costs associated with sharing of data?
   - How much should the public be told about the way in which data is collected and shared?
   - Should there be a requirement that all results be released?

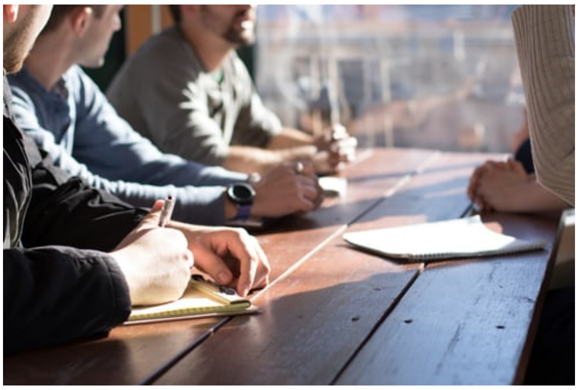

20. Your role in the citizen jury

During the citizens' jury, you will hear more about new data practices, sharing data, and **privacy** from the speakers who have expertise on particular issues and from the other participants.

You and your fellow participants will bring your own perspectives and experience to the discussion. You are not expected to be an expert on this topic.

You will be asked to discuss some of the issues related to sharing and researching linked data by **private industry**, with the other participants.

We hope that you will bring your opinions, values, and ideas about data and privacy to the **deliberation**. We encourage you to work together to make recommendations that can be used to more effectively inform **policy** decisions on data access **regulation** and **legislation.**

To facilitate discussion, we ask that you follow these ground rules:

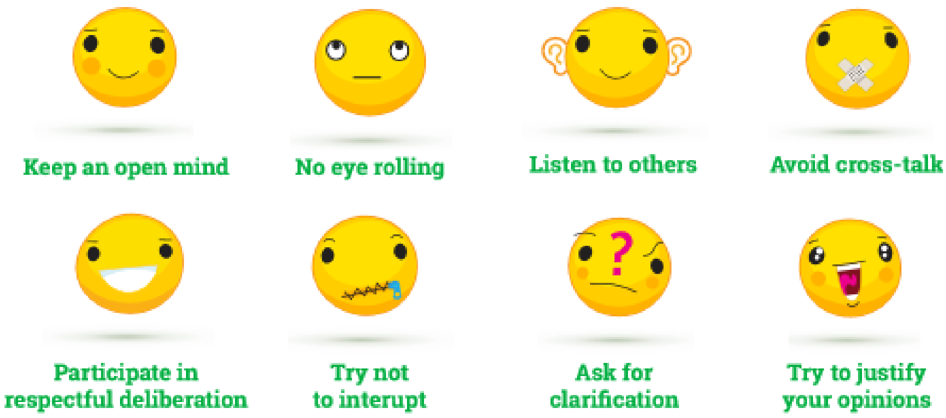

21. What happens to the findings of this jury?

We are conducting two juries, one in Parramatta and one in Wollongong. In both cases a specialist reporter will type up what is said in the juries as would happen in a court of law. The reporter will provide us with a written copy of what has been said. After both juries are completed we will take the record of what has been said and write a paper or papers which will describe what you think is important to answer the research charge. This will be published in a scientific journal which can then be made available to government agencies, disease **registries**, **data custodians**, **private industry**, advocacy and consumer groups and many others working or advocating in the area of data use and data linkage in Australia. If you wish, we can provide you access to the paper which describes this work.

In addition we will present this work at conferences and symposiums locally, nationally and internationally and to groups of interested stakeholders at local, state and national level.

We will use traditional and social media to promote the findings from this research. This may include: media releases, promotion on twitter and academic social media and sharing with a range of networks nationally and internationally.

Glossary of Terms

Administrative data: data collected in the course of providing and/or paying for services (e.g. hospital admissions, physician payment information).

Aggregated data: data which has been collected together to summarise information in a particular area. Most of the detail in the data is removed in an aggregated data set.

Algorithms: a step-by-step approach to solving a problem which is based on real data. Algorithms are usually written as a set of instructions for a computer. They allow predictions about future events which are based on information from past events.

Anonymisation: The removal of identifying details such as name and address from a data set. This is sometimes referred to as de-identification.

Autonomy: An individual's right to make decisions for themselves.

Biobank: a dedicated institution preserving biological samples (e.g., spit, blood, tissue). Biobanks also commonly store health information associated with the person from whom the sample came.

Clinical data: detailed information about specific aspects of persons, conditions and/or care (e.g. blood pressure, weight, lab results).

Consent: permission for something to happen or agreement to do something.

Criminal offence: is an act by a person or organisation (or neglecting to act where required) which is punishable by law.

Data breach: an unauthorized release of data usually through hacking or by accident.

Data custodian: a designated official responsible with approving or denying requests for access to data.

Data linkage unit: the organisations who link datasets together and create specific Linkage ID's, which allow data from different sources and organisations to be safely and securely linked together.

Data set: a collection of data that has been gathered using the same criteria or from one place e.g. all emergency room visits from a hospital or from all hospitals in one state.

Deliberation: long and careful consideration or discussion. A citizens' jury aims for informed deliberation i.e. the participants have enough information and time to consider all the relevant issues in the case under discussion.

Demographic data: is information collected from individuals such as age, gender, education level, income and employment which may then be used to describe the population.

Digital information: data kept electronically on a computer system rather than on paper.

Electronic data bases: are organized collections of data or information that are stored in computer-readable form.

Electronic health records: records relating to a single person kept in digital form by physicians or hospitals or other care providers. These records contain clinical data.

Encryption: convert (information or data) into a code, especially to prevent unauthorized access.

Facilitator: a person who helps to guide a group through a process of discussion or deliberation.

Genomic data: data which comes from the analysis of all the genetic data of a person. This is different to genetic data which tends to concentrate on single pieces of genetic material (i.e. genes).

Government health data: is data collected by government agencies in the process of providing government health services e.g. in government hospitals or in providing funding for medicines or procedures.

Human research ethics committee: a committee set up by a government, university or private agency which helps decide whether a research proposal is ethically acceptable i.e. that it meets laws and standards and that it minimises or removes real or potential harms to individuals involved in research.

Identifiers: pieces of information which on their own or in combination with other information pieces might directly identify an individual e.g. name, address, age, ethnicity.

Individual-level data: data collected that relates to a single individual.

Legislation: laws and rules made by governments.

Linked data: a collection of data, usually for research purposes, that combines two or more different sources.

Market: a place where things are bought and sold. When we talk about the market for medicines and devices we mean that once a new medicine or device has been approved it can then be legally used and sold in Australia.

Medical Benefits Schedule (MBS): is a list of the medical services for which the Australian Government will pay a Medicare rebate, to provide patients with financial help towards the costs of their medical services.

Medical imaging: the technique and process of creating detailed pictures which show or represent what is happening inside the body to help in diagnosis e.g. x-ray, CT scan, MRI.

Off label use: is the use of a medicine or medical device for a disease or condition for which it has not been approved by medical authorities. Off label use may include using a medicine or device for a particular disease or age group for which it has not been approved or giving the medicine at a higher dose than recommended or through a different type of delivery e.g. providing a medicine by injection when it has only been approved to be given by mouth.

Personal information: is information recorded about an identifiable individual. It can include: name, religion, age, marital status, blood type, home address, driver's license and any other identifying number assigned to you.

Perspectives: points of view or attitudes.

Pharmaceutical: a compound manufactured for use as a medicine. A drug used in the treatment of a disease or health condition.

Pharmaceutical benefits scheme (PBS): is a national government funded scheme which helps support the cost of medicines for all Australians. When medicines listed on the PBS are prescribed the government covers a large part of the cost so that the health consumer only pays a small portion of the cost.

Post market surveillance: is where the safety of a medicine or medical device is monitored after it has been released for sale in Australia.

Privacy: an individual's right to be free from intrusion or interference by others.

Private health providers: physicians or health providers such as GPs, dentists or physiotherapists who work as private business owners in the community.

Private industry: any privately owned business or entity, such as a corporation or private clinical practice. In this jury we are limiting our discussion to health industries involved in research and development particularly those which develop and sell medicines or medical devices.

Public interest: The public interest is the well-being and welfare of the general public and society.

Registry: Disease or patient registries are collections of data related to patients with a specific diagnosis, condition, or procedure drawn from a variety of sources.

Regulation: a rule or directive made and maintained by an authority. A regulation is created by a governmental agency, often to actually implement a given law e.g. regulations which say where tobacco can be sold and to whom.

Research: the investigation of a subject of interest or a problem.

Shareholders: persons who hold shares in a company. They may do so because they expect to benefit now or in the future from dividends on profits made by the company.

Societal issues: problems that may affect and damage society.

Therapeutic Goods Administration (TGA): is responsible for regulating therapeutic goods including medicines, medical devices, blood and blood products. The TGA is responsible for regulating the supply, import, export, manufacturing and advertising of therapeutic goods.

Trade-offs: balancing the potential benefits of any action (or inaction) with the harms which might occur from that action (or inaction).

Transparency: Transparency in dealings between businesses, institutions or government and the general public is characterised by honesty and openness.

Values: a person's values are the things that they believe are important in the way they live and work and the way that they wish society to function. People often hold many core values but some examples are honesty, reliability, loyalty, tolerance and open-mindedness.

Images Acknowledgments

Image 2 on page 26 is designed by rawpixel.com / Freepik

Images on page 11 are taken from the Parliamentary Education Office http://peo.gov.au

Image on 18 by freestocks.org on Unsplash

Image on page 19 is from https://pixabay.com and designed by OpenClipart-Vectors

Image on page 20 is by Drew Hays on Unsplash

Image on page 22 by Dylan Gillis on Unsplash

Notes …
